# Long-distance transmission of pathogenic *Vibrio* species by migratory waterbirds: a potential threat to the public health

**DOI:** 10.1038/s41598-019-52791-5

**Published:** 2019-11-08

**Authors:** Songzhe Fu, Jingwei Hao, Qian Yang, Ruiting Lan, Yi Wang, Shigen Ye, Ying Liu, Ruijun Li

**Affiliations:** 1 College of Marine Technology and Environment, Dalian Ocean University, Dalian, China; 2 College of Fisheries and Life Science, Dalian Ocean University, Dalian, China; 3 Center for Microbial Ecology and Technology, Ghent University, Coupure Links 653, Gent, Belgium; 4 School of Biotechnology and Biomolecular Sciences, University of New South Wales, Sydney, New South Wales Australia

**Keywords:** Phylogenetics, Water microbiology

## Abstract

A potential mechanism for the global distribution of waterborne pathogens is through carriage by the migratory waterbirds. However, this mode of transmission has yet been confirmed epidemiologically. Here, we conducted whole genome sequencing of *Vibrio* spp. collected from waterbirds, sediments, and mollusks in the estuary of the Liaohe River in China to investigate this transmission mode. We found that a *V. parahaemolyticus* strain isolated from a waterbird was clonally related to the other *V. parahaemolyticus* strains obtained from the sediments and mollusks, and three *V. mimicus* strains isolated from bird feces were genomically related to those found in the mollusks and upstream groundwater, suggesting that the bird-carried Vibrio strains were acquired through the direct predation of the local mollusks. Surprisingly, two bird-carried *V. parahaemolyticus* strains belonging to the same clone were identified in Panjin and Shanghai, which are over 1,150 km apart, and another two were found at two locations 50 km apart, further supporting that waterbirds are capable of carrying and disseminating these pathogens over long distances. Our results provide the first evidence of direct transmission from mollusks to waterbirds and confirm that waterbirds act as disseminating vehicles of waterborne pathogens. Effective surveillance of migratory waterbirds along their routes will be valuable for predicting future epidemics of infectious diseases.

## Introduction

*Vibrio parahaemolyticus* is a Gram-negative comma-shaped bacterium and a leading cause of seafood-related gastroenteritis^1^. Infection by *V. parahaemolyticus* is often associated with the consumption of raw or undercooked seafood^2^. In the USA alone, *V. parahaemolyticus* is estimated to cause approximately 35,000 human illnesses each year^3^. *V. parahaemolyticus* serogroup O3:K6 has been recognized to cause pandemic-level disease and has been intensively investigated^4^. The serogroup emerged in India in 1996 and was rapidly spread to other Asian countries and other continents^4^. In South America, O3:K6 was first reported in Peru in 1996^5^ and then rapidly spread to Chile in 1997 and the USA in 1998, to Brazil in 2001 and to Mexico in 2004^6^.

*V. parahaemolyticus* and other *Vibrio* species have been found widely in various environments, including estuaries and rivers. Extensive studies have identified the natural reservoirs of *V. parahaemolyticus* and other *Vibrio* species^7,8^. Invertebrates such as chironomids, copepods, and oysters are suggested to be the natural reservoirs for the *Vibrio* species. Evidence of the transmission of *Vibrio* species from fish to great cormorants has also recently been published^9^.

As early as the 1970s, pathogenic *Vibrio* species were isolated from the faces of various waterbird species, including *Ardea herodias* (great blue heron) and *Larus delawarensis* (ridge-billed gull)^10^. However, the public health significance of waterbird-carried *Vibrio* species has not attracted the deserved attention of the scientific community, and the avenue through which the waterbirds acquire the pathogens remains poorly understood. Laviad-Shitrit *et al*.^11^ found that both *Vibrio* species and *Aeromonas* species were abundant in many types of birds in Israel, suggesting that migratory waterbirds are potential disseminators of waterborne pathogens. However, the roles that southward autumn and northward spring bird migrations might play in the transmission of waterborne pathogens and the evolutionary origins of these pathogens have rarely been examined. There have been no molecular epidemiological studies of the origins of waterbird-carried *Vibrio* species. Whether migratory birds mediate the spread of these pathogens remains largely unknown.

**Figure 1:**
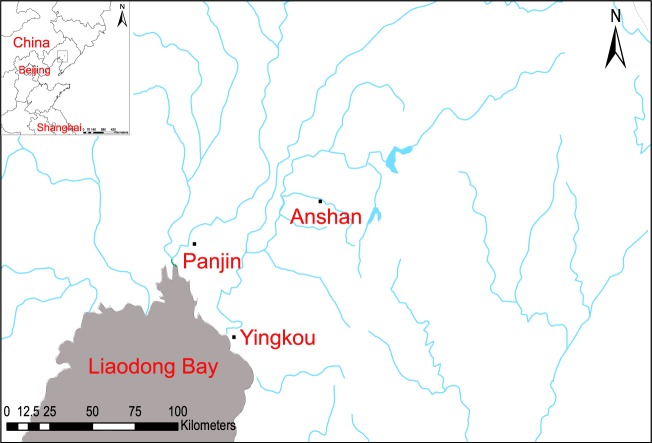
Sampling sites in this study. The sampling positions in Anshan, Panjin, and Yingkou are indicated in the square. The sampling sites were mapped by the ArcGIS Desktop 10.2 software (http://desktop.arcgis.com/).

Yingkou and Panjin are located along the estuary of the Liaohe River in North China (Fig. 1). In 2017, we isolated several multidrug-resistant (MDR) *Vibrio* species from fish farms in the upstream reaches of the Liaohe River (Fig. 1). To evaluate the impact of the aquacultural activities on the downstream river, we sampled sediments, mollusks, and waterbird feces in Panjin and Yingkou. Unexpectedly, abundant *V. parahaemolyticus* and other pathogens were identified in the waterbird feces and mollusks. In this study, we investigated the evolutionary origins of the *Vibrio* species carried by waterbirds using whole-genome sequencing.

## Results

### Epidemiological investigation and identification of waterborne bacterial pathogens on fish farms

In August 2017, massive outbreaks of ornamental fish disease occurred on several fish farms in Anshan. An epidemiological investigation showed that all the sampled fish farms bred their own brood fish, without importing of parent fish from other sources, which excluded the possible input of pathogens from external sources. A total of 60 fish feed samples from three fish farms were analyzed microbiologically but no *Vibrio* sp. was isolated. Groundwater was the only source of the water used for aquaculture. The fish farmers pumped the groundwater into their aquaculture systems without any treatment or disinfection because groundwater is usually considered to be of high quality suitable for aquaculture. Three *Vibrio mimicus* strains were isolated from fish and groundwater in a fish farm (Table 1). A follow-up investigation showed that several tons of wastewater from fish ponds were discharged into the river after the clean-up of the outbreak-related ponds, which might have resulted in the transmission of the pathogens from Anshan into the estuary downstream. Antibiotics, sulfamethoxazole, florfenicol, fluoroquinolone, sulfonamide, and tetracycline were used widely for the treatment of fish diseases in these farms. Concerns were raised on antibiotic resistance development as the outbreaks occurred despite the use of various antibiotics to treat the infections.

**Table 1:** General features of strains isolated in this study.

Sampling site	Sources	Species	Strain Name	Isolation date	Sequence type
Anshan	Fish	*V. mimicus*	VM14	Oct-17	/
	Underground water	*V. mimicus*	VM34	Mar-18	/
	Underground water	*V. mimicus*	VM61	Jun-18	/
Yingkou	Mollusk	*V. parahaemolyticus*	YK38	Oct-17	ST1823
	Sediment	*V. parahaemolyticus*	YK39	Oct-17	ST1823
	Sediment	*V. parahaemolyticus*	YK40	Oct-17	ST1823
	Sediment	*V. parahaemolyticus*	YK13	Oct-17	ST2006
	Sediment	*V. parahaemolyticus*	YK17	Oct-17	ST2007
	Mollusk	*V. parahaemolyticus*	YK41	Oct-17	ST1823
	Mollusk	*V. parahaemolyticus*	YK32	Oct-17	ST236
	Mollusk	*V. parahaemolyticus*	YK34	Oct-17	ST1498
	Mollusk	*V. mimicus*	VM20	Mar-18	/
	Mollusk	*V. parahaemolyticus*	YK51	Jun-18	ST415
	Mollusk	*V. parahaemolyticus*	YK45	May-18	ST1823
	Bird feces (Common Greenshank)	*V. parahaemolyticus*	YK33	Oct-17	ST2008
	Bird feces (Common Greenshank)	*V. scophthalmi*	YK47	Oct-17	/
	Bird feces (Eurasian Curlew)	*V. parahaemolyticus*	YK49	Oct-17	ST1823
	Bird feces (Black-tailed Godwit)	*V. mimicus*	VM27	Apr-18	/
	Bird feces (Black-tailed Godwit)	*V. mimicus*	VM37	Apr-18	/
	Bird feces (Black-tailed Godwit)	*V. mimicus*	VM41	Apr-18	/
Panjin	Estuary	*V. parahaemolyticus*	PJ42	Oct-17	ST1823
	Estuary	*V. parahaemolyticus*	PJ43	Oct-17	ST1823
	Sediment	*V. parahaemolyticus*	PJ44	Oct-17	ST1823
	Bird feces (Common Greenshank)	*V. parahaemolyticus*	PJ37	Oct-17	ST2008
	Bird feces (Common Greenshank)	*V. parahaemolyticus*	PJ18	Oct-17	ST415
	Bird feces (Common Greenshank)	*V. parahaemolyticus*	PJ35	Oct-17	ST3
Shanghai	Bird feces (Common Greenshank)	*V. parahaemolyticus*	SH50	Oct-18	ST415

### Isolation of *vibrio* species in the estuary of liaohe river

To investigate the impact of wastewater discharge on the river downstream, especially on a migratory waterbird sanctuary, we conducted a field investigation of the downstream river delta region. A total of 180 bird fecal samples were collected in the cities of Shanghai, Yingkou and Panjin (Table S1). Mollusk and sediment samples were also collected from the intertidal zones at Yingkou and Panjin. The monitoring of the environmental variables in Anshan, Yingkou and Panjin showed that seawater in Yingkou had highest concentration of dissolved organic carbon (22.3 mg L^−1^), total phosphorus (67 μg L^−1^), and nitrite (0.45 mg L^−1^) (Table S2).

In total, 27 strains of *Vibrio* species were isolated in this study, 70.4% of which were *V. parahaemolyticus*. In Yingkou, 10 *V. parahaemolyticus* strains and one *V. mimicus* strain were isolated from the sediment and mollusk samples, whereas two strains of *V. parahaemolyticus* were recovered from bird feces. In addition, three *V. mimicus* strains and one *V. scophthalmi* strain were also isolated from bird feces.

In Panjin, three *V. parahaemolyticus* strains were isolated from bird feces, and another three were isolated from sediments or estuary seawater. In Shanghai, one *V. parahaemolyticus* strain was isolated out of 25 bird feces sampled.

### Phylogenetic and evolutionary analysis of *V. parahaemolyticus* from bird feces

To determine the origins of *V. parahaemolyticus* in bird feces, we sequenced the 19 *V. parahaemolyticus* strains (Table S3). *In silico* multilocus sequence typing (MLST) of the *V. parahaemolyticus* strains identified eight sequence types (STs) among the 19 strains. Nine strains isolated from mollusks, estuary and sediments from Panjin and Yingkou were all assigned to ST1823, whereas the strains PJ18, YK51, PJ35, YK32, YK34, YK13, and YK17were identified as ST415, ST415, ST3, ST236, ST1498, ST2006 and ST2007, respectively (Table 1). YK33 and PJ37 both belonged to ST2008. In addition, the SH50 strain obtained from Shanghai was also identified as ST415.

All except three new STs were found in the PubMLST database with various frequencies. ST3 is a recognized pandemic ST with the highest frequency in the database (249/2887, 8.63%). ST1823, ST415, ST236 and ST1498 were presented in the database with much lower frequencies of 0.48%, 1.5%, 0.07% and 0.1%, respectively (Table S4).

We further selected the publicly available genomes for phylogenetic and evolutionary analyses based on two criteria. First, the genomes of the strains phylogenetically close to the sequenced strains on MLST typing were selected. Second, the strains that occurred geographically close to the sampling sites or at sites overlapping bird migration routes were selected for comparison.

**Figure 2:**
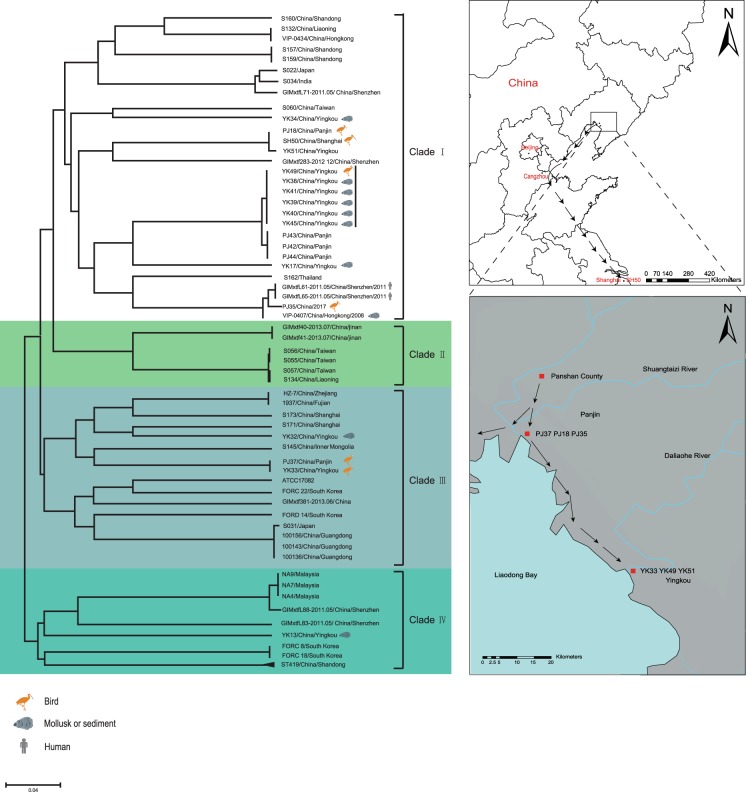
Geophylogeny of *V. parahaemolyticus* genomes and the migration route of the common greenshank from Panjin to Yingkou in autumn. left: maximum likelihood phylogenies of 66 *V. parahaemolyticus* genomes. Bootstrapping was performed with 1,000 replicates. Right: The isolation sites of bird-carried *V. parahaemolyticus*. The sampling sites were mapped by the ArcGIS Desktop 10.2 software (http://desktop.arcgis.com/). Precise migration route of the common greenshank from Panjin to Yingkou in autumn 2017 is indicated in arrows.

Finally, we incorporated 47 publicly available genomes for the phylogenetic analysis to examine the origins of our strains (Table S5). The recombinant SNPs were removed by the recombination detection program (RDP) as described in the supplemental file (Table S6, Appendix 1). Only non-recombinant core genome SNPs were used for phylogenetic inference. The ST1823 genome tree is shown in Fig. 2. The nine ST1823 strains from Yingkou and Panjin formed two separate clusters differing by 77 SNPs (Fig. S1), indicating that they belonged to two different lineages but might share a most recent common ancestor circulating in the river delta region. Notably, strain YK49, which was isolated from a bird (Eurasian curlew), differed by two SNPs from the other ST1823 strains isolated from mollusks or sediments in Yingkou (Fig. S1). Strikingly, two bird-carried ST415 strains, PJ18 and SH50 (differing by ten SNPs), were found in Panjin and Shanghai (over 1100 km apart), respectively. These two strains differ by 1,129 SNPs from another ST415 strain, YK51, which was obtained from the mollusks in Yingkou. In addition, two bird-carried strains, YK33 and PJ37, differing by two SNPs, were also isolated from the same species of waterbirds (common greenshank) in two different locations 50 km apart, which intersect the migration route of the common greenshank (Fig. 2). Other mollusk-derived strains were dispersed throughout the genome tree (Fig. 2).

**Figure 3:**
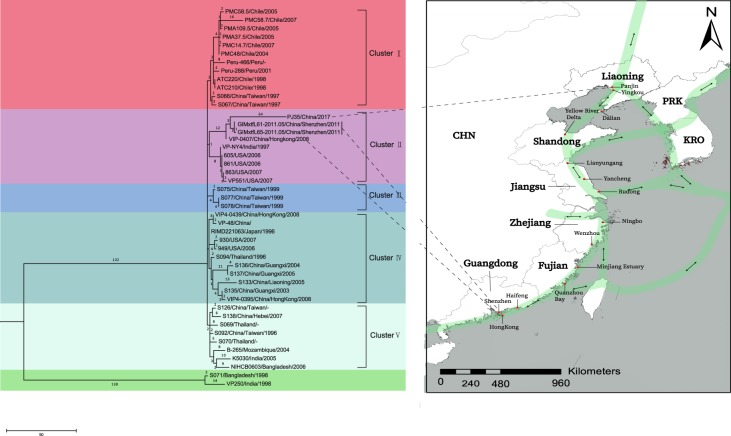
The divergence of 44 ST3 *V. parahaemolyticus* genomes. Left: maximum parsimony tree of 44 ST3 *V. parahaemolyticus* genomes. Homoplasy index (HI) was 0.0 for the maximum parsimony tree. The numbers above the branches indicate the numbers of SNPs. Right: migration route of the common greenshank, with stopovers at Shenzhen, Haifeng, Quanzhou Bay, Minjiang Estuary, Rudong, Yancheng, Lianyungang, Yellow River Delta, Yingkou, and Panjin. The sampling sites were mapped by the ArcGIS Desktop 10.2 software (http://desktop.arcgis.com/).

For the sole ST3 strain, PJ35, isolated from a waterbird in Panjin, we included 44 other ST3 genomes in the analysis (Fig. 3). The phylogenetic tree showed that PJ35 was genomically most closely related to the two Shenzhen strains and one Hong Kong strain with 64 strain-specific SNPs.

### Origins of *V. mimicus* from waterbird feces

**Figure 4:**
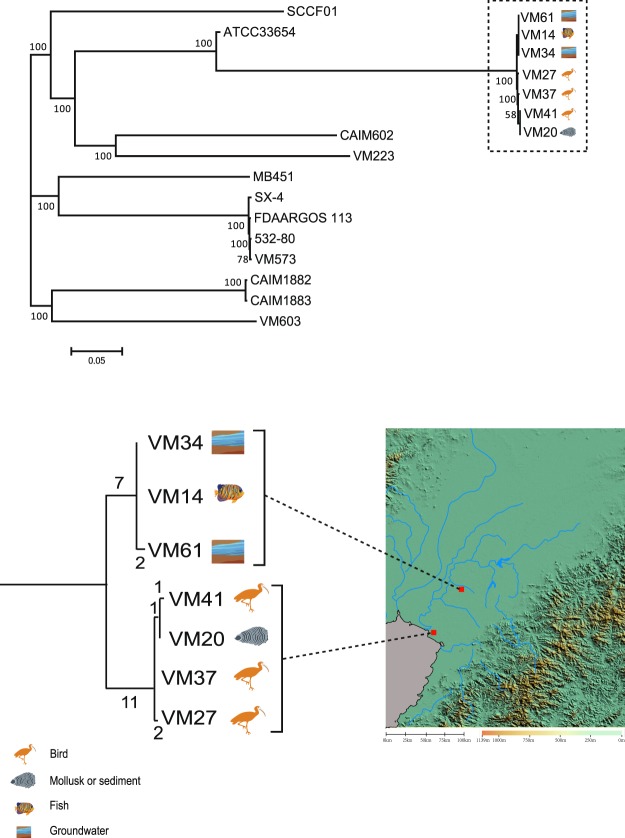
Phylogenetic relationships of *V. mimicus* strains. Top: Maximum-likelihood phylogenies of 24 *V. mimicus* strains. The bootstrap was performed with 1000 replicates. The unit of the scale bar indicates the evolutionary distance in substitutions per nucleotide. Down: Geophylogeny of seven sequenced *V. mimicus* strains. The numbers above the branches indicate the numbers of SNPs. Locations of the strains obtained from Anshan and Yingkou are indicated on the right side. The satellite imagery of the investigated region was created by Global Mapper v20 (http://www.bluemarblegeo.com/products/global-mapper.php).

We next determined the phylogenetic relationships of the seven *V. mimicus* strains from this study and 12 *V. mimicus* sequences from the publicly available genomes (Table S7). The three bird-carried *V. Mimicus* strains clustered with those isolated from sediments or groundwater from the same area (Fig. 4).

The four Yingkou and the three Anshan strains were closely related, differing by only up to 18 SNPs, indicating that they might share a most recent common ancestor (Fig. 4). The strains VM14, VM34, and VM61 (from Anshan) belonged to the same cluster which was repeatedly isolated from underground water, with seven branch-specific SNPs. The strains VM20, VM27, VM37, and VM41 (from Yingkou) formed another cluster with 11 branch-specific SNPs.

### Antibiotic resistance genes (ARGs) in the sequenced strains

Genomic analysis confirmed that most of the *V. parahaemolyticus* strains carried *blaCARB-47* (except for YK13 with a *blaCARB-48* gene). A few of the sequenced *V. parahaemolyticus* strains carried additional ARGs, such as *dfrA31* and *qnrC*. Seven sequenced *V. mimicus* strains from birds and underground water all carried the resistance genes *aph(3*″*)-Ib*, *aph(6)-Id*, *qnrVC4*, *floR*, *sul2*, *tet*(59), and *dfrA6* on their chromosomes (Table S8).

### Modeling the dynamics of the *V*. *parahaemolyticus*-carrying birds

We used the intertidal zone of the estuary of the Liaohe River in Yingkou as the site for modelling the dynamics of the *V. parahaemolyticus*-carrying birds.

To set the modeling parameters, the numbers of common greenshank that stopped over in the investigated region were counted every week. To estimate the sizes of the prey population(mollusks), the dynamics of mollusk density in the sediment were determined with the method described by Finke *et al*.^12^.

The prevalence of *V. parahaemolyticus* in mollusks (∏) was estimated by an equation with three parameters (see Eq. (1) in Methods): the total pool size (total number of mollusks in all investigated sampling sites), the number of positive pools, and the number of pools tested. In our case, the number of pools tested was 138. As all of the samples came from six different sampling times (each time consisted of 25 sampling points) and the average number of mollusks at each sampling point was 660 individuals, the size of the pools was 6 × 25 × 660 = 99,000. Because six *V*. *parahaemolyticus* strains were isolated from the mollusks, the number of positive pools was six. When we input these parameters into Eq. 1, the prevalence of *V*. *parahaemolyticus*-infected mollusks was calculated to be 4.5 × 10^−7^ (95% confidence interval: 1.64 × 10^−7^ to 9.77 × 10^−7^).

We also observed the dynamics of the mollusks and the waterbirds (common greenshank) populations from August 2017 to November 2017. As shown in Fig. S2A, there was a slight decline in the population density of the mollusks during this period, after which it was maintained at approximately 660 individuals/m^2^. To simplify the model, we assumed that the total biomass of the mollusks was nearly constant. At the end of August, there were only a few hundred common greenshanks in Yingkou; the population peaked at approximately 1,300 birds, which then gradually left Yingkou after October 2017. The number of common greenshanks that arrived and stayed in Yingkou showed a nearly normal distribution (Fig. S2B). Therefore, these data allowed us to predict the dynamics of the *V*. *parahaemolyticus*-carrying common greenshank using Eq. (2) (see Methods). Overall, the *V*. *parahaemolyticus*-carrying common greenshank population remained at a relatively low level, with a maximum of 95 individuals predicted to carry *V*. *parahaemolyticus* in the studied region.

## Discussion

### Genomic analysis of the bird-carried *Vibrio* species confirmed that migratory birds are disseminators of *Vibrio* species

Using high-resolution genome sequencing, we determined the precise origin of *Vibrio* species in birds. Our results demonstrated that several *Vibrio* strains isolated from bird feces were clonally related to the strains found in the mollusks in the local area. Therefore, the role of migratory birds in the transmission of *Vibrio* species must be reevaluated. The dissemination of *Vibrio* species by migratory birds is rarely considered^13^, and very little is known about the role that migratory birds play in these processes. Although it has recently been recognized that the dissemination of antibiotic resistance can be mediated by migratory birds^14^, the potential of migratory birds to disseminate *Vibrio* species had been neglected by the scientific community for more than three decades, until 2008 in a study by Halpern *et al*. (2008) which suggested that the dispersal of *Vibrio* species by waterbird may be attributable to their direct predation of chironomids and copepods^15^. Pretzer *et al*. (2017) also reported that many *V. cholerae* isolates from European countries were genetically related to the strains present in a European lake (Neusiedler See)^16^. Therefore, they speculated that the long-distance transfer of *V. cholerae* strains was probably via migratory birds. However, this mode of transmission lacked solid epidemiological evidence. In this study, a *V. parahaemolyticus* ST3 strain was isolated from a fecal sample of a common greenshank that was genomically closely related to two human clinical strains from Shenzhen (South China) and one strain isolated from an oyster in Hong Kong. The finding provides an exceptional clue for the pandemic expansion of ST3 *V. parahaemolyticus* populations across the globe. Since it was first identified in Japan (where it was spread from Indonesia), the mode through which this pandemic strain spread to China and arrived on the American continent has remained a matter for speculation. Many studies have suggested that the transmission of *Vibrio* populations can be mediated by the movement of ocean currents or human activities^5^. However, the movement of ocean currents is in the opposite direction to the course of the epidemic expansion in the American continent (Fig. S3). The explosive spread of the ST3 clone over hundreds of kilometers of coastline along the Pacific coast of South America since 1996 also cannot be explained simply by human activity^16^. The identification of this ST3 strain and other STs in bird feces suggests that the transmission of *V. parahaemolyticus* by migratory birds is another important factor, filling the gaps in the hypothesis described above.

In addition, the isolation of the *V. parahaemolyticus* ST1823 strains from both bird feces (Eurasian curlew) and mollusks also provides solid evidence that bird predation of local mollusks is an important source of *Vibrio* species. In addition, two closely related *V. parahaemolyticus* strains, YK33 and PJ37, were also isolated from a migratory bird (common greenshank) in two locations 50 km distant apart.

Another striking finding was that two bird-carried ST415 strains, PJ18 and SH50, were found in Panjin and Shanghai over 1100 km apart, respectively, further supporting the proposition that waterbirds mediates the long-distance transmission of *V. parahaemolyticus*. In addition, these two strains were genomically related to another ST415 strain which was obtained from the mollusks. The pubmlst data showed that the ST415 strain was not commonly identified in North China, the origin of ST415 *V. parahaemolyticus* in Yingkou remains unknown. One plausible possibility is that bird feces containing ST415 strains contaminated the seafood in Yingkou, resulting in its detection in the mollusks. Therefore, this ST could become a pandemic clone and poses a potential threat to human health. Because many *V. parahaemolyticus* STs (such as ST36) have recently been shown to be transmitted across countries, it is likely that some of these STs will be carried by migratory birds to become pandemic clones. Modeling the dynamics of *V*. *parahaemolyticus*-carrying birds also indicated that the presence of *V*. *parahaemolyticus* in birds, albeit infrequently, is likely to contaminate *V*. *parahaemolyticus*-free seafood and thus poses a potential threat to public health.

In this study, we also identified an extraordinary example of how *V. mimicus* strains can be moved between distant areas. *Vibrio mimicus* isolates were repeatedly isolated from underground water in the upstream reaches of the Liaohe River, which are probably the sources of contamination of the river delta region in Yingkou. More importantly, we found the same *V. mimicus* clone in the underground water in Anshan and in bird feces in Yingkou, which directly supported the hypothesis that the predation of mollusks and zooplankton by migratory birds played an essential role in the dissemination of *V. mimicus*.

### Limitations of this study

One of the limitations of this study was that we only collected samples from three major waterbird species. Apart from the common greenshank, black-tailed godwit, and Eurasian curlew, other waterbird species were also abundant at the sampling sites, including the marsh sandpiper, common sandpiper, common redshank, common snipe, and Eurasian oystercatcher, all of which are long-distance migratory birds that depend on mollusks as their main food source. Therefore, the transmission of *Vibrio* species from mollusks to birds is probably a general model for most waterbird and the total carriage of pathogens by waterbirds is likely to be much higher. Another limitation of this study was that we did not collect enough samples nationally to reconstruct the spatial and temporal transmission patterns of bird-carried *V. parahaemolyticus*. To what extent this mode of transmission contributes to the global dissemination of *V. parahaemolyticus* and other *Vibrio* species remains to be assessed. In future studies, we will investigate the prevalence of *Vibrio* species at other sites along the migration routes.

Very few *Vibrio* species was isolated from the fish and water sampled in this study. This might be associated with the methodology used for the enrichment of *Vibrio* species. As our main purpose was to obtain *Vibrio* species from bird feces, water samples were not filtered, which might not have recovered vibrios living in zooplankton. In addition, the numbers of fish samples were too small to recover enough *Vibrio* species. However, previous studies suggested that fish might be the nature reservoirs and vectors of *Vibrio* spp.^9^, and waterbirds can be contaminated with vibrios by feeding on fish^17^. Further studies need to focus on the epidemiological links between *Vibrio* species in waterbirds and *Vibrio* species in contaminated fish.

### Public health significance of waterbird migration

**Figure 5:**
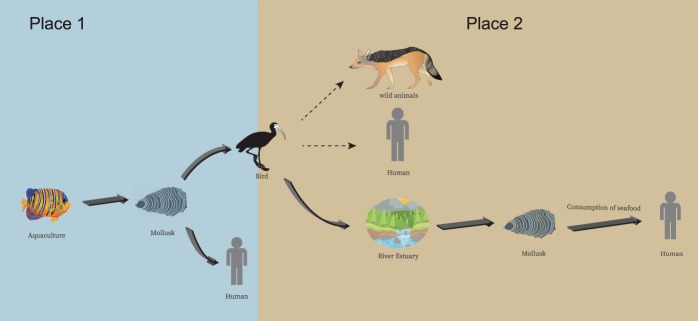
Possible spread of *Vibrio* species from mollusks to migratory waterbird.

Mollusks, crustaceans, crabs, and oysters are the common food resources of waterbirds, and the majority of waterbird species are long-distance migratory birds. Millions of birds in Liaoning travel between their winter homes (extending from South China to Australia) and their summer breeding grounds (Siberia) each year, and the stopover sites in these migrations include most coastal regions of China, the Korean Peninsula, and Japan. For instance, the common greenshank is widely distributed from the UK to Siberia as its breeding ground. They stop over at Panjin and Yingkou from late August to early November and return to their winter grounds in Australia and Southeast Asia. Thus, the migratory common greenshank might acquire *Vibrio* species through predation along its migration route and carry them to its next destination. The identification of another ST415 strain in Yingkou also highlighted that mollusks were the source of the *Vibrio* species isolated from the feces of the waterbirds. Bird feces containing these pathogens would contaminate the environment and seafood at the next destination and thus pose a potential threat to human health. This large-scale migration occurs twice a year, constantly promoting the dissemination of pathogens. During this process, these birds may also be caught by wild animals or humans, leading to further pathogen spread (Fig. 5).

The findings of this study provide solid support for the hypothesis that *Vibrio* species are potentially spread across countries and continents by rivers and the migration of birds. From a public health perspective, the migration of waterbirds across national and intercontinental borders provides a mechanism for the global dispersal of bacterial pathogens.

The results herein also identified antibiotic-resistance genes (ARGs) in waterborne pathogens collected from the fish farms. The development of MDR may be associated with the overuse of these antibiotics in aquaculture. The ARGs were likely transferred by waterborne pathogens along the river. Interestingly, we found that the MDR *V. mimicus* strains originating from Anshan had established their population in the estuary of the Liaohe River and been transmitted to the waterbird-inhabited stopover sites, which confirmed a dissemination pattern of ARGs from the upstream river to the estuary and subsequently transferred to birds. The findings underscore that the aquacultural activity in the upstream river may greatly promote the spread of ARGs and trigger a chain reaction of antibiotic resistance transfer in the ecological systems in the estuary region. Moreover, because migratory birds may carry MDR bacteria and transport them to regions far from their original habitats, multidrug resistance is also transmitted, albeit infrequently, to places that traditionally have no public awareness of disease prevention^17^.

The roles of wild birds in the spread of avian flu and tick-borne viruses are well recognized^18,19^. Therefore, the migration of birds is probably a general route of pathogen transmission. This requires global attention. To prevent the rapid spread of pathogens, public health authorities must strengthen their active surveillance of birds and monitor their exact migration routes. Based on the information presented here, the public should reduce their direct and indirect contact with birds in the migration season and minimize the potential exposure of birds to these pathogens.

## Conclusions

Our study suggest that the migration of birds is a significant mechanism in the long-distance dissemination of *V. parahaemolyticus* and *V. mimicus*. Waterbirds may be the missing link in understanding the long-distance transmission of bacterial pathogens across continents. These findings confirm that bacterial pathogens can be transmitted from mollusks to migratory birds in estuaries. They also extend our understanding of the mechanisms underlying the long-distance spread of bacterial pathogens, which is very important in the prevention of pandemics. Effective surveillance of waterbirds should be undertaken to monitor the dissemination of waterborne pathogens along their migration routes, which will also provide valuable information for predicting future epidemics of infectious diseases.

## Materials and Methods

### Sampling

From August 30 to October 27, 2017, and from March 13 to April 24, 2018, 66 water samples were collected from fish farms in Anshan and the nearby groundwater (Fig. 1). At the same time, in-field observations were conducted in the intertidal zones at Panjin, Yingkou and Shanghai to capture the moment of excretion for individual birds and record the bird species. A total of 180 samples of fresh feces from seven types of birds, including bean goose (*Anser fabalis*), white-front goose (*Anser erythropus*), Saunders’s Gull (:*Larus saundersi*), red-billed gull (*Chroicocephalus novaehollandiae scopulinus*), common greenshank (*Tringa nebularia*), Eurasian curlew (*Numenius arquata*) and black-tailed godwit (*Limosa limosa*) were collected (Table S1); and 170 sediment samples and 77 mollusk samples were collected from Panjin and Yingkou. All the samples were sent to the laboratory within 12 h of collection. In addition, a range of environmental variables (water temperature, pH, total phosphorus, dissolved organic carbon, and nitrite) were determined according to Kirschner *et al*.^20^.

### Isolation and identification of the *Vibrio* species in water, sediment, and bird fecal samples

The sediments, bird feces and water samples were aseptically disaggregated and precultured in alkaline peptone water at 37 °C for 12 h. Afterwards, one loop of culture was spread directly in triplicate onto thiosulfate citrate bile salts (TCBS) agar plates and incubated at 37 °C for 24 h. The yellow and green colonies on the TCBS, which were suspected to be *Vibrio* species, were subcultured in Luria broth (LB) to produce pure cultures. For bacterial identification, a partial sequence of 16 S rRNA was amplified with PCR using the primers 27 F (5′-AGAGTTTGATCCTGGCTCAG-3′) and 1492 R (5′-GGTTACCTTGTTACGACTT-3′)^21^. The amplicons with a size of approximately 1400 bp were sequenced with Sanger sequencing technology at Shanghai Jingtong Biotech Co. Ltd. The similarity analysis of the 16S rRNA gene sequence was performed using the BLASTn program on NCBI (https://blast.ncbi.nlm.nih.gov/Blast.cgi) to confirm the bacterial species.

### Genome sequencing and *de novo* assembly

Genomic DNA was extracted from overnight bacterial cultures grown ontrypticase soy agar, and fragmented and tagged for multiplexing with the Nextera XT DNA Sample Preparation Kit (Illumina). The tagged DNAs were sequenced with the Illumina HiSeq. 2500 platform at Beijing Novogene Bioinformatics Technology Co., Ltd. The FASTQ reads were quality trimmed with Trimmomatic (v0.36)^22^, and bases with a PHRED score of <30 were removed from the trailing end. To obtain the draft genomes, the contigs were assembled *de novo* with SPAdes version 3.0^23^.

### *In silico* MLST

*In silico* MLST of *V. parahaemolyticus* was performed with the MLST 1.8 server at the Center for Genomic Epidemiology (https://cge.cbs.dtu.dk//services/MLST/)^24^.

### Identification of SNPs and phylogenetic inferences

The genomes of ATCC 33654, and RIMD221063 were used as the reference genomes to call the SNPs for *V. mimicus*, and *V. parahaemolyticus*, respectively. A total of 47 publicly available *V. parahaemolyticus* genomes were obtained from GenBank. Using these and those of the 27 sequenced strains, stringent SNP calling was performed with a custom pipeline, as described previously, to guarantee that only genuine SNPs were included in the analysis^25^. The *V. parahaemolyticus* core genome SNPs were extracted based on the previously defined *V. parahaemolyticus* core genome^26^. Recombination events were confirmed with RDP3^27^. Each recombinant site was confirmed with at least three different analytical methods. Fifteen *V. mimicus* genomes available from GenBank were also used to investigate the evolutionary relationships of the *V. mimicus* strains sequenced in this study (Table S5). The phylogenetic trees were constructed with the maximum likelihood method using RAxML 7.2.8^28^. Generalized Time Reversible (GTR) with Gamma rates (G) and Invariant sites (I) (GTR + G + I) model was used. For closely related genomic strains, maximum parsimony algorithms were used to precisely identify the SNPs that differed among them, in PAUP 4.0^29^.

### Gene content analysis of *V. mimicus* and *V. parahaemolyticus*

RAST sever was used to annotate the sequences of each genome determined with next-generation sequencing^30^. ARGs were identified with ResFinder^31^.

### Modeling the dynamics of *V. parahaemolyticus*-carrying birds

We used the intertidal zone of Yingkou in the estuary of the Liaohe River as the location for modeling the dynamics of *V. parahaemolyticus*-carrying birds.

To set the modeling parameters, the numbers of common greenshank stopover points in the investigated region were counted every week. To estimate the size of the prey population (mollusks), the dynamics of the mollusk density in the sediment were determined with the method described by Finke GR *et al*.^13^.

If we let *k* be the pool size, *x* the number of positive pools, and *m* the number of pools tested, and the prevalence of *V. parahaemolyticus*-infected mollusks is denoted ∏, the MLE of *V. parahaemolyticus* prevalence in mollusks (∏) can be described as suggested by Cowling *et al*.^32^ as follows:

1 $${\hat{\pi }}_{MLE}=1-{(1-\frac{x}{m})}^{1/k}.$$

The details of the modeling inference can be found in the Supplemental File (Appendix 2).

If we let H and B(t) be the sizes of the prey population (mollusks) and the predator population (common greenshank), respectively, the number of *V. parahaemolyticus*-infected birds can be described as follows:

2 $${\rm{\beta }}({\rm{t}})={P}\times {s}\times {\rm{H}}\times {\rm{B}}({\rm{t}})$$

where *P* denotes the prevalence of *V. parahaemolyticus* in the mollusks, and*s* indicates the daily predation rate. If we assume that *P* and *s* are constant values, then *s* × H is the number of mollusks that can be caught by one bird daily, and *P* × *s* × H is the number of *V. parahaemolyticus*-infected mollusks that can be caught by one bird per day.

## Supplementary information

SUPPLEMENTARY INFO

## Data Availability

The raw sequencing data were submitted to GenBank (NCBI) under the BioProject No. PRJNA496566.
