# Supplementary material for: Long-distance transmission of pathogenic Vibrio species by migratory waterbirds: a potential threat to the public health
Source: Sci Rep. 2019 Nov 8;9:16303. doi: 10.1038/s41598-019-52791-5 (PMC6841736; doi:10.1038/s41598-019-52791-5)
Supplement: Supplementary file 1 — SUPPLEMENTARY INFO [file 41598_2019_52791_MOESM1_ESM.pdf]

Supplemental material

Long-distance transmission of pathogenic *Vibrio* species by migratory waterbirds: a potential threat to the public health

Songzhe Fu[1#], Jingwei Hao[2#], Qian Yang[3#], Ruiting Lan[4], Yi Wang[2], ShigenYe[2], Ying Liu[1], and Ruijun Li[2]

[#] contribute equally to this paper

1. College of Marine Technology and Environment, Dalian Ocean University, Dalian, China
2. College of Fisheries and Life Science, Dalian Ocean University, Dalian, China
3. Yellow Sea Fisheries Research Institute, Chinese Academy of Fishery Sciences
4. School of Biotechnology and Biomolecular Sciences, University of New South Wales, Sydney, New South Wales, Australia

*** Correspondence:**

Songzhe Fu

fusongzhe@hotmail.com

Ruijun Li

liruijun@dlou.edu.cn

Supplementary Figure

Figure S1 Maximum-parsimony tree of ST1823 *V. parahaemolyticus* genomes. Homoplasy index (HI) is 0.0. The tree was rooted using strain YK17. The number above the branches indicates the number of SNPs.

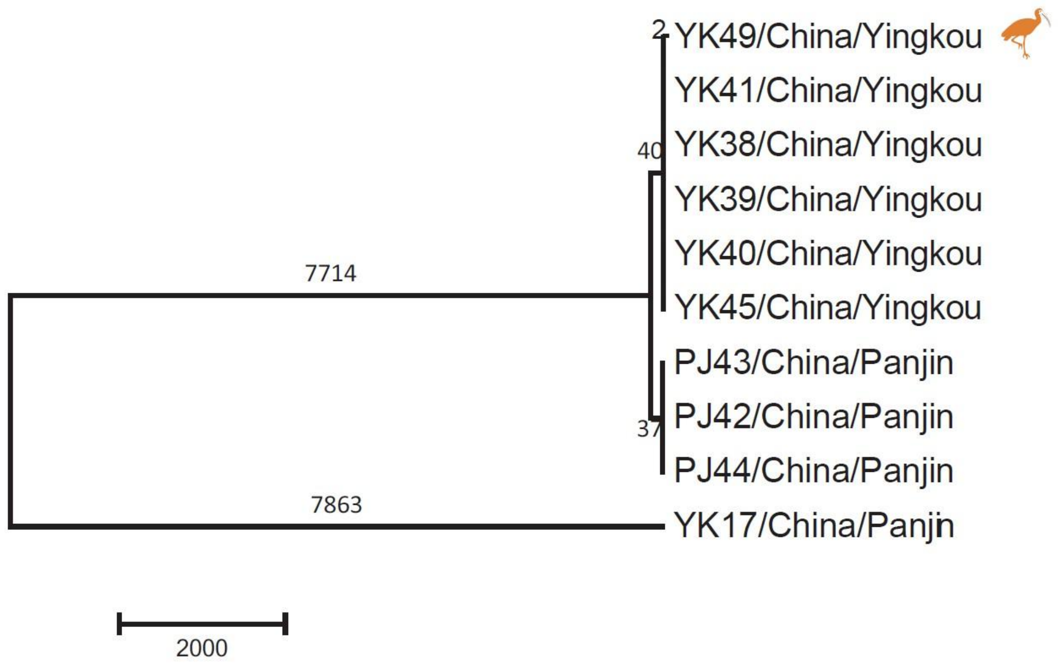

Figure S2 Dynamics of mollusk density in the intertidal zone of Yingkou (A) and modeling of the dynamics of *V. parahaemolyticus* (VP)-carrying birds (B). The solid line indicates the observed number of Common Greenshank, while dot line presents the predicted number of VP-carrying Common Greenshank.

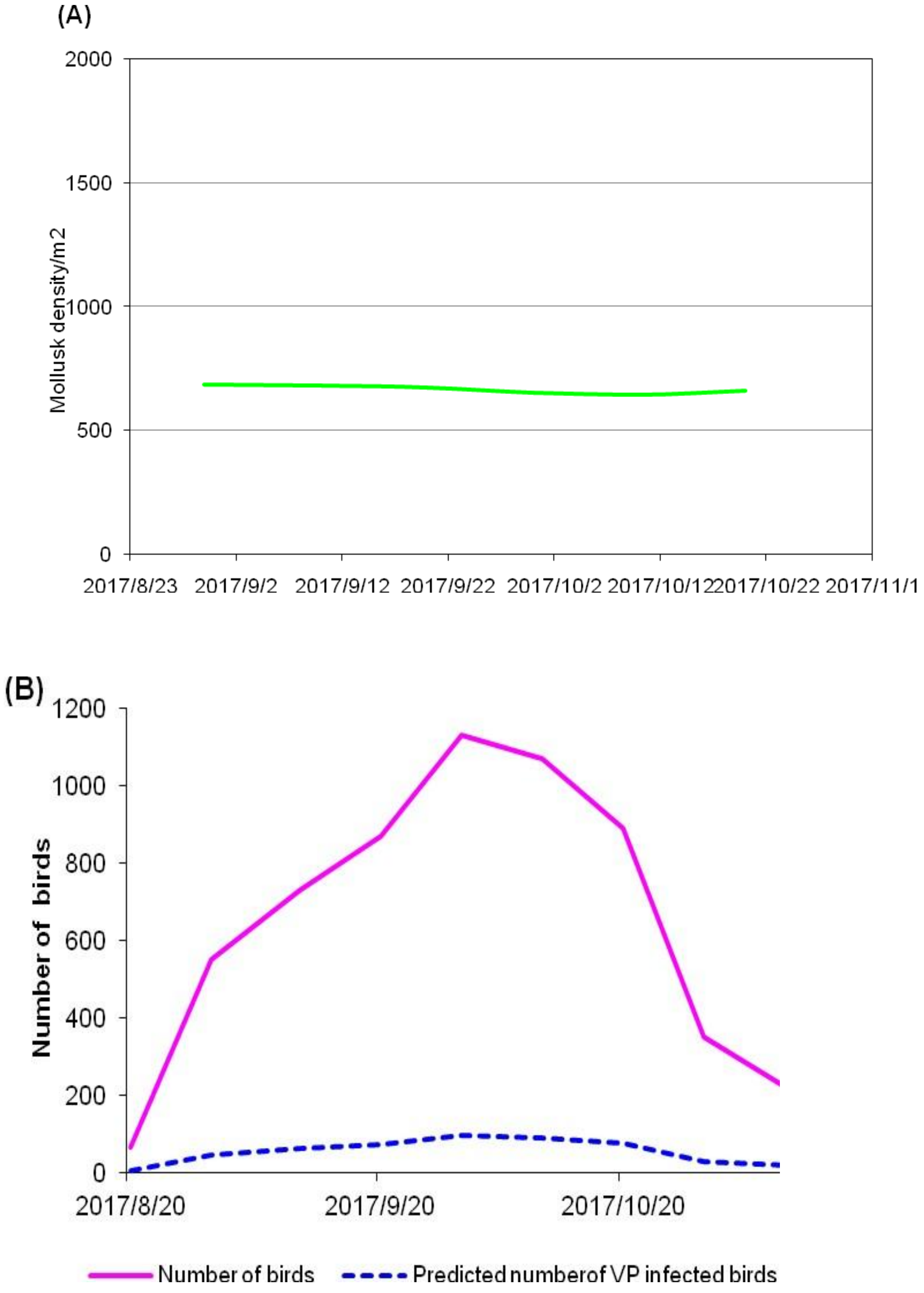

Figure S3 Distribution of the *V. parahaemolyticus* isolates belonged to ST3 and its correlation with global bird migration routes. The map was generated by the ArcGIS Desktop 10.2 software (http://desktop.arcgis.com/). The eight major bird migration routes around the global are labeled in eight colored circles as suggested by Yun et al. (2015)[1]. Blue and purple arrow indicates the direction of warm and cold currents in the Pacific Ocean, respectively. Red regions in the global map indicate the countries or regions with the clinical cases of *V. parahaemolyticus* ST3 isolates between 1996 and 2013 as suggested by Nair et al.(2007) [2]and Velazquez-Roman et al.(2013)[3].

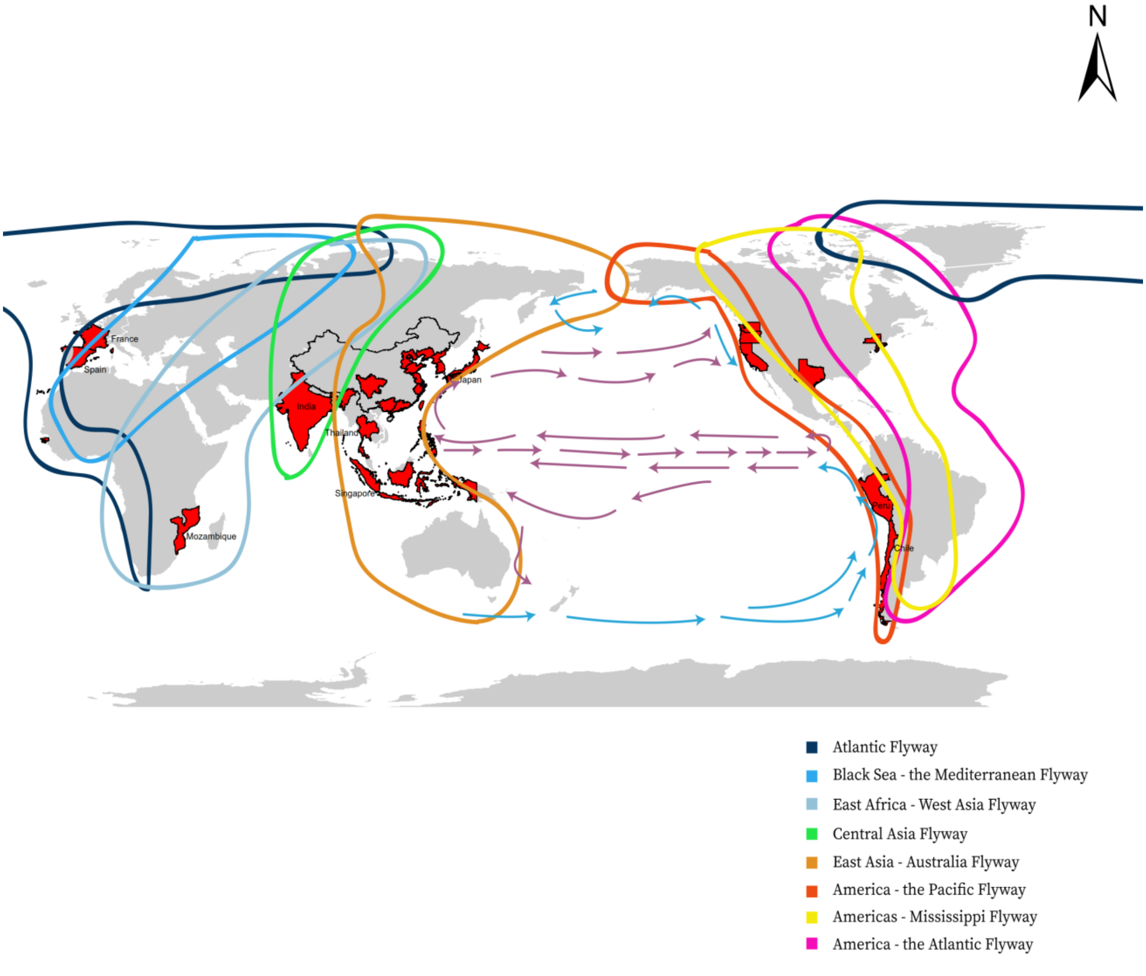

Supplementary text

Appendix 1

Detection of recombination in *V. parahaemolyticus* (VP)

Previous studies suggested that recombination was the major factor driving the evolution of the *V. parahaemolyticus* [4]. To remove the potential impact of recombination, RDP program[5] was employed to identify recombination events to obtain a more accurate phylogenetic reconstruction.

SNP analysis identified 102,306 *V. parahaemolyticus* core genome SNPs. As the accessory genomes have been removed from the core genome of VP, the number of recombination events was relatively lower than the values reported by another study[4]. RDP identified 12 large recombination events in the core genome of VP (Table S5), many of which were also reported previously [4]. Some hotspots of recombination sites, including the genes involved in the ABC transporter substrate-binding protein, secretion protein, DNA binding (peptidases), endonuclease, metalloendopeptidase, and N(6)-L-threonylcarbamoyladenine synthase activities were also identified in this study.

A total of 11,613 recombinant SNPs were removed, which contributed to11.3% of the variants identified in the dataset. For the ST3 strains, a total of 613 recombinant SNPs were removed, which contributed to 0.0468% of the variants identified in the core genome of *V. parahaemolyticus*. Only non-recombinant core genome SNPs were used to infer the genomic relationship of *V. parahaemolyticus.* Maximum parsimony (MP) method was used to infer the phylogenetic relationship of ST3 clones by using non-recombinant core genome SNPs. Only one MP tree was generated.

Appendix 2

Modeling the dynamics of VP-carrying birds by a predator-prey model

We chose the intertidal zone near the Yingkou City for sampling and modeling, which has a square with approximate 1.5×10^6 m^2

If the prevalence is extremely low (<0.1%), a reasonable approach might be to assume that there is only one positive sample in each positive pool.

Let k be the pool size, x the number of positive pools, and m the number of pools tested. The prevalence of VP-infected mollusk was denoted as ∏.

As suggested by Cowling et al. (1999) [5], the maximum-likelihood estimator (MLE) of

VP prevalence in mollusk (∏) can be written as

$$\hat{\pi}_{MLE} = 1 - \left(1 - \frac{x}{m}\right)^{1/k} \qquad (1)$$

Note that x (the number of positive pools) has a binomial distribution with parameters: n and P.

The variance of MLE can be estimated by Eq. (2)

$$\widehat{Var(\hat{\pi}_{MLE})} = \frac{(x/m)(1-x/m)^{2/k-1}}{k^2 m} \qquad (2)$$

In our case, the number of pools tested was 138. As all of samples came from six times of samplings (each time consists of 25 sampling points) and average number of mollusk in each sampling point was 660 individuals, the size of the pools was $6 \times 25 \times 660 = 99{,}000$. Because six *V. parahaemolyticus* strains were isolated from mollusks, the number of positive pools was six.

When we input these parameters into equation 1, the prevalence of *V. parahaemolyticus*-infected mollusks was calculated to be 4.5 $\times 10^{-7}$ (95% confidence interval: 1.64–9.77 $\times 10^{-7}$).

The number of infected of VP-carrying birds can be estimated by the following equations:

$\beta(t)=P \times s \times H \times B(t)$ (3)

P denotes the prevalence of VP in the mollusk, while *s* indicates the daily predation rate. B(t) is the total number of birds in the investigated region in certain date. If we assume *P* and *s* are the constant values, then $s \times H$ is the number of mollusks can be caught by one bird daily, where $P \times s \times H$ indicates the number of VP infected mollusks that caught by a bird per day. The predation rate *s* can be calculated as：

S= the number of mollusks predated per day/total number of mollusks presented in the places bird stopover. (4)

Based on our in-field observation, we found that Common Greenshank typically stopover at around 180-500 m^2 sediment for predation daily (average 200 m^2). The average density of mollusks was around 660 individual/ m^2. Each individual usually consumes 20-30 mollusks per day. Therefore, the predation rate *s* can be calculated as

$s = 25/(200 \times 660) = 1.9 \times 10^{-4}$.

As the square of the investigated area in Yingkou is 1.5×10^6 m^2,
the total number of mollusk can be calculated as

H= density of mollusks $\times$square of investigated area=$660 \times 1.5 \times 10^6 = 9.9 \times 10^8$

Therefore, we can calculate the number of infected of VP-carrying birds by Eq. (4):

$\beta(t)= P \times s \times H \times B(t)= 4.45 \times 10^{-7} \times 1.9 \times 10^{-4} \times 9.9 \times 10^8 \times B(t) = 0.084 \times B(t)$

Thus, we can obtain the dynamics of VP infected birds. As we do not know the chance of VP to pass the digestive tract, the predicted number of VP infected birds might be overestimated.

Supplementary table

Table S1 Information of the water, sediment and bird feces sampled in the bird migration season

	Sample type		Sampling site[a]			
			Anshan	Yingkou	Shanghai	Panjing
Water			66	5	0	8
Sediment			/	34	0	20
Mollusk			/	138	0	32
Crab			/	5	0	25
bird feces	Sandpipper	Common Greenshank	/	41	25	21
		Black-billed Godwit	/	24	0	12
		Eurasian Curlew	/	10	0	0
	Gull	Black-billed Gull	/	8	0	12
		Red-billed Gull	/	1	0	8
	Goose	Bean Goose	/	5	0	3
		White-front Goose	/	6	0	4

a: the numbers in the table indicates the number of individual samples

Table S2 Environmental variables of three sampling sites in the sampling period

Sampling site			pH	Temp(℃)[a]	DOC(mg L^{-1})[b]	Total phosphorus(μg L^{-1})	Nitrite(mg L^{-1})
Anshan	groundwater	Average	8.2	17.5	11.2	45	0.31
		Min	8	13.7	10.6	36	0.45
		Max	8.4	21.3	23.1	79	0.21
Yingkou	seawater	Average	8.3	18.3	22.3	67	0.45
		Min	7.9	12.3	11.2	58	0.24
		Max	8.7	22.7	28.7	87	0.76
Panjin	seawater	Average	8.6	18.2	11.9	23	0.24
		Min	8.1	11.8	10.9	18	0.15
		Max	8.9	22.6	15.2	39	0.48

a. Water Temperature

b. Dissolved organic carbon

Table S3 General features of genomes sequenced in this study

Species	Strain Name	N50[a]	Contig Number	Total Length (bp)	GC content(%)	Accession
V.mimicus	VM14	175236	60	4148181	46.7	SAMN10233604
V.mimicus	VM34	673223	51	4165063	46.7	SAMN10233608
V.mimicus	VM61	175640	58	4154324	46.7	SAMN10233642
V.parahaemolyticus	YK38	539419	56	5381751	45.1	SAMN10233644
V.parahaemolyticus	YK39	539460	57	5375477	45.1	SAMN10239529
V.parahaemolyticus	YK40	539459	56	5374470	45.1	SAMN10239530
V.parahaemolyticus	YK13	317677	65	5128954	45.2	SAMN10239531
V.parahaemolyticus	YK17	430667	48	5149308	45.3	SAMN10239532
V.parahaemolyticus	YK41	539271	12	5114506	45.2	SAMN10239612
V.parahaemolyticus	YK32	980059	24	5059557	45.3	SAMN10240008
V.parahaemolyticus	YK34	532654	35	5217375	45.2	SAMN10240007
V.parahaemolyticus	YK45	539460	59	5375779	45.1	SAMN10240009
V.mimicus	VM20	521068	53	4172938	46.7	SAMN10240010
V.parahaemolyticus	YK33	557374	13	4952683	45.4	SAMN10240051
V.scophthalmi	YK47	151923	52	4699994	44.6	SAMN10240052
V.parahaemolyticus	YK51	185265	87	5127397	44.6	SAMN11475395
V.parahaemolyticus	YK49	214972	14	3924830	44.7	SAMN10240056
V.mimicus	VM27	175640	67	4166494	46.7	SAMN10240057
V.mimicus	VM37	662907	48	4169986	46.7	SAMN10240079
V.mimicus	VM41	672907	53	4170794	46.7	SAMN10240080
V.parahaemolyticus	PJ42	653087	58	5568010	45.1	SAMN10240082
V.parahaemolyticus	PJ43	539460	56	5575081	45.1	SAMN10240083
V.parahaemolyticus	PJ44	539418	61	5572779	45.1	SAMN10240085
V.parahaemolyticus	PJ37	557374	38	5117397	45.3	SAMN10240148
V.parahaemolyticus	PJ18	177983	108	5380834	45.3	SAMN10240086
V.parahaemolyticus	PJ35	425693	44	5093812	45.3	SAMN10240147
V.parahaemolyticus	SH50	675840	35	5120439	45.3	SAMN11475416

a:a median value of contig lengths for de novo assemblies

Table S4 General information of four STs in the pubmlst data

Strain	collection_date	country	isolation_source	serotype
**ST415**				
LN_Vp274	2016	China: Dalian	environmental	
962	2015	Philippines	environmental	
GZ96	2014	China: Guangzhou	environmental	
GZ97	2014	China: Guangzhou	environmental	
642B3	2013	China	environmental	O12
VP13-009	2013	China	clinical	O3:KUT
E74	2013	Thailand	environmental	
E76	2013	Thailand	environmental	
2012-163	2012	China: Zhejiang	environmental	
3/24	2012	Ecuador	environmental	
2012-170	2012	China: Zhejiang	environmental	
I-172	2012	Ecuador	environmental	
CR053-18	2012	USA	environmental	
SH11VP246	2011	China: Shanghai	environmental	O10
907	2011	Ecuador	environmental	
S6-b	2011	China	environmental	
IT_CR_Vp274	2011	Venetian	environmental	
2011-00142-02	2011	Canada	environmental	
Vp061	2011	China	environmental	O2:Kunk
IT_CR_Vp79	2011	Venetian	environmental	
XM-VP185	2010	China	environmental	
IB3884	2004	Mozambique	clinical	O3:K58
IB3885	2004	Mozambique	clinical	O3:K58
IB3891	2004	Mozambique	clinical	O3:K58
IB3886	2004	Mozambique	clinical	O3:K58
VP142	2003	Thailand	environmental	Ounk:Kunk
VP168	1996	Thailand	clinical	O3:Kunk
**ST1823**				
PH-1096	2015	Philippines	environmental	
PH-1042	2015	Philippines	environmental	
2014W310	2014	China: Zhejiang	environmental	
2014W298	2014	China: Zhejiang	environmental	
VP1043B1	2013	China	environmental	O4
ningbo2012-044	2012	China: Zhejiang	environmental	O5:KUT
41-4-i	2011	Madurankuliya	environmental	
23-3-ii	2011	Puttalam	environmental	
VP06157	2006	China	clinical	O8:K22
VP130A	2003	Thailand	environmental	O1:Kunk
VP130B	2003	Thailand	environmental	O1:Kunk
VP12	1999	Thailand	environmental	O1KUT
**ST236**				
YK32	2017	China: Liaoning	environmental	
VP26	1999	Thailand	environmental	
**ST1498**				
YK34	2017	China: Liaoning	environmental	
III-132	2012	Ecuador	environmental	
VN-4016	2010	Netherland	environmental	

Table S5 General features of *V. parahaemolyticus* used in this study

Strain	Country province(or city)		Year	isolation source	ST
S160	China	Shandong	2006	environment	ST329
S132	China	Liaoning	2005	human	ST332
VIP-0434	China	Hongkong	2008	human	ST332
S157	China	Shandong	2006	environment	ST1009
S159	China	Shandong	2006	environment	ST1009
S022	Japan	/	1984	human	ST8
S034	India	/	1999	human	ST8
GIXMxtfL71-2011.05	China	Shenzhen	2011	human	ST8
S060	China	Taiwan	1992	human	ST272
GIXMxtf283-2012	China	Shenzhen	2012	human	Unknown
S162	Thailand	/	2007	environment	ST595
GIXMxtfL61-2011.05	China	Shenzhen	2011	human	ST3
GIXMxtfL65-2011.05	China	Shenzhen	2011	human	ST3
VIP-0407	China	Hongkong	2008	mollusk	ST3
GIXMxtf40-2013.07	China	Jinan	2013	human	ST396
GIXMxtf41-2013.07	China	Jinan	2013	human	ST396
S056	China	Taiwan	1994	human	ST69
S055	China	Taiwan	1994	human	ST69
S057	China	Taiwan	1994	human	ST69
S134	China	Liaoning	2005	human	ST527
HZ-7	China	Zhejiang	2014	human	ST150
PB1937	China	Fujian	2012	human	ST150
S173	China	Shanghai	2005	environment	ST289
S171	China	Shanghai	2007	environment	ST288
S145	China	Inner Mongolia	2006	human	Unkonwn
ATCC17082	Japan	/	2005	human	ST1
FORC22	South Korea	/	2017	human	ST233
GIXMxtf381-2013.06	China	/	2013	human	Unkonwn
FORC14	South Korea	/	2015	human	ST1629
S031	Japan	/	1984	human	ST189
100156	China	Guangdong	2013	human	Unknown
100143	China	Guangdong	2013	human	Unknown
100136	China	Guangdong	2008	human	Unknown
NA9	Malaysia	/	2017	Shrimp	ST1911
NA7	Malaysia	/	2017	Shrimp	ST1911
NA4	Malaysia	/	2017	Shrimp	ST1911
GIXMxtfL88-2011.05	China	Shenzhen	2011	human	Unknown
GIXMxtfL83-2011.05	China	Shenzhen	2011	human	Unknown
FORC8	South Korea	/	2014	human	ST984
FORC18	South Korea	/	2014	human	ST984
S154	China	Shandong	2005	human	ST419
S156	China	Shandong	2005	human	ST419
S166	China	Shandong	2005	human	ST419
S144	China	Shandong	2005	human	ST419
S158	China	Shandong	2005	human	ST419
S153	China	Shandong	2005	human	ST419
S151	China	Shandong	2005	human	ST419

Table S6. List of core genes of *V. parahaemolyticus* in strain RIMD 2210633 and recombination sites identified

locus	associated with recombnation	gene product
VP0001	NO	flavodoxin
VP0003	NO	inner membrane protein translocase component YidC
VP0004	NO	ribonuclease P
VP0005	NO	50S ribosomal protein L34
VP0006	YES	amino acid ABC transporter ATP-binding protein
VP0007	YES	amino acid ABC transporter permease
VP0012	YES	DNA polymerase III subunit beta
VP0015	YES	hypothetical protein
VP0016	YES	hypothetical protein
VP0018	YES	16 kDa heat shock protein A
VP0019	NO	valine--pyruvate transaminase
VP0022	NO	glycyl-tRNA synthetase subunit alpha
VP0025	YES	sulfur transfer protein SirA
VP0026	YES	hypothetical protein
VP0032	YES	potassium uptake protein TrkH
VP0034	YES	DNA-binding transcriptional regulator IlvY
VP0035	NO	ketol-acid reductoisomerase
VP0036	NO	hypothetical protein
VP0038	NO	multidrug resistance protein
VP0040	NO	TetR family transcriptional regulator
VP0041	NO	ATP-dependent DNA helicase Rep
VP0042	NO	cytochrome c5
VP0044	NO	hypothetical protein
VP0045	NO	hypothetical protein
VP0047	YES	peptide ABC transporter ATP-binding protein
VP0048	YES	peptide ABC transporter periplasmic peptide-binding protein
VP0050	YES	peptide ABC transporter permease
VP0055	NO	RNA polymerase ECF-type sigma factor
VP0056	NO	hypothetical protein
VP0061	NO	multidrug transmembrane resistance signal peptide protein
VP0069	NO	hypothetical protein
VP0070	NO	oligopeptidase A
VP0072	NO	DNA-binding transcriptional regulator AsnC
VP0073	NO	hypothetical protein
VP0074	NO	hypothetical protein
VP0075	NO	MadN protein
VP0076	NO	universal stress protein A
VP0077	NO	ferritin
VP0078	NO	universal stress protein UspB
VP0079	NO	hypothetical protein
VP0082	NO	transmembrane protein
VP0083	NO	hypothetical protein
VP0086	NO	hypothetical protein
VP0087	NO	hypothetical protein
VP0089	NO	hypothetical protein
VP0093	NO	hypothetical protein
VP0094	NO	hypothetical protein
VP0095	NO	ubiquinone/menaquinone biosynthesis methyltransferase
VP0096	NO	hypothetical protein

VP0097	NO	ubiquinone biosynthesis protein UbiB
VP0098	NO	twin arginine translocase protein A
VP0100	NO	hypothetical protein
VP0103	NO	hypothetical protein
VP0104	NO	delta-aminolevulinic acid dehydratase
VP0105	NO	hypothetical protein
VP0107	NO	DNA polymerase I
VP0109	NO	ribosome biogenesis GTP-binding protein YsxC
VP0110	NO	cytochrome c4
VP0113	NO	hypothetical protein
VP0114	NO	hypothetical protein
VP0115	NO	coproporphyrinogen III oxidase
VP0116	NO	adenosine deaminase
VP0117	NO	GGDEF family protein
VP0119	NO	nitrogen regulation protein NR(II)
VP0120	NO	hypothetical protein
VP0121	NO	glutamine synthetase
VP0122	NO	BipA protein
VP0123	NO	hypothetical protein
VP0124	NO	hypothetical protein
VP0130	NO	Hsp33-like chaperonin
VP0131	NO	hypothetical protein
VP0132	NO	general secretion pathway protein C
VP0134	NO	general secretion pathway protein E
VP0135	NO	general secretion pathway protein F
VP0136	NO	general secretion pathway protein G
VP0137	NO	general secretion pathway protein H
VP0141	NO	general secretion pathway protein L
VP0142	NO	general secretion pathway protein M
VP0143	NO	general secretion pathway protein N
VP0144	NO	CysQ protein
VP0145	NO	ADP-ribose diphosphatase NudE
VP0146	NO	DNA uptake protein
VP0147	NO	ComF-like protein
VP0148	NO	BioH protein
VP0149	NO	hypothetical protein
VP0150	NO	hypothetical protein
VP0151	NO	hypothetical protein
VP0152	NO	transcription elongation factor GreB
VP0155	NO	osmolarity sensor protein
VP0156	NO	xanthine/uracil permease family protein
VP0157	NO	ATP-dependent DNA helicase RecG
VP0158	NO	tRNA guanosine-2'-O-methyltransferase
VP0160	NO	DNA-directed RNA polymerase subunit omega
VP0161	NO	guanylate kinase
VP0164	NO	TonB system transport protein ExbD2
VP0165	NO	TonB system transport protein ExbB2
VP0166	NO	TolR
VP0167	NO	hypothetical protein
VP0170	NO	transmembrane ABC transporter protein
VP0172	NO	binding protein component of ABC transporter
VP0173	NO	oligopeptide ABC transporter ATP-binding protein

VP0174	NO	oligopeptide ABC transporter ATP-binding protein
VP0175	NO	hypothetical protein
VP0177	NO	ribonuclease PH
VP0178	NO	orotate phosphoribosyltransferase
VP0179	NO	lipid A biosynthesis lauroyl acyltransferase
VP0180	NO	nucleoid occlusion protein
VP0181	NO	bifunctional phosphopantothenoylcysteine decarboxylase/phosphopantothenate synthase
VP0185	NO	50S ribosomal protein L28
VP0186	NO	50S ribosomal protein L33
VP0239	NO	triosephosphate isomerase
VP0240	NO	5-carboxymethyl-2-hydroxymuconate delta isomerase
VP0241	NO	hypothetical protein
VP0242	NO	hypothetical protein
VP0243	NO	hypothetical protein
VP0244	NO	fructose 1,6-bisphosphatase II
VP0246	NO	hypothetical protein
VP0251	NO	cell division protein FtsN
VP0253	NO	primosome assembly protein PriA
VP0254	NO	hypothetical protein
VP0255	NO	50S ribosomal protein L31
VP0256	NO	30S ribosomal protein S10
VP0257	NO	50S ribosomal protein L3
VP0258	NO	50S ribosomal protein L4
VP0259	NO	50S ribosomal protein L23
VP0260	NO	50S ribosomal protein L2
VP0261	NO	30S ribosomal protein S19
VP0262	NO	50S ribosomal protein L22
VP0263	NO	30S ribosomal protein S3
VP0264	NO	50S ribosomal protein L16
VP0265	NO	50S ribosomal protein L29
VP0266	NO	30S ribosomal protein S17
VP0267	NO	50S ribosomal protein L14
VP0268	NO	50S ribosomal protein L24
VP0269	NO	50S ribosomal protein L5
VP0270	NO	30S ribosomal protein S14
VP0271	NO	30S ribosomal protein S8
VP0272	NO	50S ribosomal protein L6
VP0273	NO	50S ribosomal protein L18
VP0274	NO	30S ribosomal protein S5
VP0275	NO	50S ribosomal protein L30
VP0276	NO	50S ribosomal protein L15
VP0277	NO	preprotein translocase subunit SecY
VP0278	NO	50S ribosomal protein L36
VP0279	NO	30S ribosomal protein S13
VP0280	NO	30S ribosomal protein S11
VP0281	NO	30S ribosomal protein S4
VP0282	NO	DNA-directed RNA polymerase subunit alpha
VP0283	NO	50S ribosomal protein L17
VP0284	NO	hypothetical protein
VP0285	NO	FKBP-type peptidylprolyl isomerase
VP0286	NO	hypothetical protein
VP0287	NO	ElaA protein

VP0288	NO	ATP-dependent RNA helicase DbpA
VP0290	NO	bifunctional 2',3'-cyclic nucleotide 2'-phosphodiesterase/3'-nucleotidase periplasmic protein
VP0291	NO	uroporphyrin-III C-methyltransferase
VP0292	NO	sulfate adenylyltransferase
VP0293	NO	sulfate adenylyltransferase
VP0296	NO	adenylylsulfate kinase
VP0297	NO	hypothetical protein
VP0299	NO	hypothetical protein
VP0300	NO	hypothetical protein
VP0301	NO	ABC transporter ATP-binding protein
VP0303	NO	hypothetical protein
VP0305	NO	hypothetical protein
VP0306	NO	methionine sulfoxide reductase A
VP0307	NO	hypothetical protein
VP0308	NO	hypothetical protein
VP0309	NO	hypothetical protein
VP0310	NO	hypothetical protein
VP0311	NO	inorganic pyrophosphatase
VP0313	NO	fructose-1,6-bisphosphatase
VP0314	NO	UDP-N-acetylmuramate:L-alanyl-gamma-D-glutamyl-m eso-diaminopimelate ligase
VP0316	NO	thiamine transporter substrate binding subunit
VP0317	NO	thiamine transporter membrane protein
VP0318	NO	thiamine ABC transporter ATP-binding protein
VP0320	NO	hypothetical protein
VP0323	NO	hypothetical protein
VP0324	NO	arginine repressor ArgR
VP0325	NO	malate dehydrogenase
VP0326	NO	hypothetical protein
VP0327	NO	octaprenyl-diphosphate synthase
VP0329	NO	50S ribosomal protein L27
VP0330	NO	GTPase ObgE
VP0331	NO	hypothetical protein
VP0334	NO	diadenosine tetraphosphatase
VP0335	NO	ApaG protein
VP0336	NO	dimethyladenosine transferase
VP0338	NO	survival protein SurA
VP0339	NO	organic solvent tolerance protein
VP0340	NO	Dna-J like membrane chaperone protein
VP0341	NO	hypothetical protein
VP0342	NO	isopropylmalate isomerase small subunit
VP0344	NO	3-isopropylmalate dehydrogenase
VP0346	NO	2-isopropylmalate synthase
VP0347	NO	hypothetical protein
VP0348	NO	hypothetical protein
VP0350	NO	leucine transcriptional activator
VP0351	NO	long-chain-fatty-acid-CoA ligase
VP0352	NO	acetolactate synthase 3 catalytic subunit
VP0353	NO	acetolactate synthase 3 regulatory subunit
VP0354	NO	sensory box/GGDEF family protein
VP0355	YES	LuxZ
VP0356	YES	pyruvate kinase
VP0357	YES	hypothetical protein

VP0358	YES	DeoR family transcriptional regulator
VP0359	YES	glucosamine--fructose-6-phosphate aminotransferase
VP0369	YES	mannitol-1-phosphate 5-dehydrogenase
VP0370	YES	PTS system mannitol-specific transporter subunit IIABC
VP0374	YES	hypothetical protein
VP0378	YES	hypothetical protein
VP0404	YES	RNA polymerase sigma factor RpoD
VP0406	YES	hypothetical protein
VP0408	YES	DNA-binding/iron metalloprotein/AP endonuclease
VP0409	YES	beta-ketoadipate enol-lactone hydrolase
VP0411	YES	dihydroneopterin aldolase FolB
VP0412	NO	2-amino-4-hydroxy-6- hydroxymethyldihydropteridine pyrophosphokinase
VP0413	NO	undecaprenyl pyrophosphate phosphatase
VP0414	NO	multifunctional tRNA nucleotidyl transferase/2'3'-cyclic phosphodiesterase/2'nucleotidase/phosphatase
VP0415	NO	general secretion pathway protein A
VP0419	NO	hypothetical protein
VP0420	NO	hypothetical protein
VP0424	NO	bifunctional heptose 7-phosphate kinase/heptose 1-phosphate adenyltransferase
VP0426	NO	MutT/nudix family protein
VP0427	NO	hypothetical protein
VP0429	NO	esterase
VP0430	NO	DNA topoisomerase IV subunit B
VP0431	NO	DNA topoisomerase IV subunit A
VP0432	NO	protease DegS
VP0433	NO	protease Do
VP0434	NO	cytochrome d ubiquinol oxidase subunit III
VP0436	NO	hypothetical protein
VP0438	NO	50S ribosomal protein L13
VP0441	NO	ubiquinol-cytochrome c reductase, iron-sulfur subunit
VP0442	NO	ubiquinol-cytochrome c reductase, cytochrome b
VP0443	NO	ubiquinol-cytochrome c reductase, cytochrome c1
VP0444	NO	stringent starvation protein A
VP0449	NO	lipoprotein
VP0450	NO	hypothetical protein
VP0451	NO	hypothetical protein
VP0452	NO	S-adenosyl-methyltransferase MraW
VP0453	NO	cell division protein FtsL
VP0454	NO	penicillin-binding protein 3
VP0455	NO	UDP-N-acetylmuramoylalanyl-D-glutamate--2, 6-diaminopimelate ligase
VP0456	NO	UDP-N-acetylmuramoylalanyl-D-glutamyl-2, 6-diaminopimelate-D-alanyl-D-alanyl ligase
VP0457	NO	phospho-N-acetylmuramoyl-pentapeptide- transferase
VP0458	NO	UDP-N-acetylmuramoyl-L-alanyl-D-glutamate synthetase
VP0459	NO	cell division protein FtsW
VP0461	NO	UDP-N-acetylmuramate--L-alanine ligase
VP0463	NO	cell division protein FtsA
VP0464	NO	cell division protein FtsZ
VP0465	NO	UDP-3-O-[3-hydroxymyristoyl] N-acetylglucosamine deacetylase
VP0466	NO	hypothetical protein
VP0467	NO	preprotein translocase subunit SecA
VP0468	NO	mutator MutT protein
VP0469	NO	dihydrodipicolinate reductase
VP0470	NO	carbamoyl phosphate synthase small subunit

VP0475	NO	LysR family transcriptional regulator
VP0476	NO	hypothetical protein
VP0477	NO	hypothetical protein
VP0478	NO	adenosylcobinamide-phosphate synthase
VP0479	NO	5'-methylthioadenosine/S-adenosylhomocysteine nucleosidase
VP0481	NO	glutamate synthase subunit beta
VP0484	NO	glutamate synthase, large subunit
VP0485	NO	hypothetical protein
VP0486	NO	sensory box/GGDEF family protein
VP0487	NO	aerobic respiration control sensor protein ArcB
VP0488	NO	DNA polymerase III, beta chain
VP0492	NO	ribonuclease activity regulator protein RraA
VP0493	NO	hypothetical protein
VP0494	NO	bifunctional aspartokinase I/homoserine dehydrogenase I
VP0495	NO	homoserine kinase
VP0496	NO	threonine synthase
VP0499	NO	hypothetical protein
VP0500	NO	uracil-DNA glycosylase
VP0502	NO	hypothetical protein
VP0503	NO	sodium/alanine symporter
VP0504	NO	hypothetical protein
VP0505	NO	ATP-dependent RNA helicase SrmB
VP0506	NO	hypothetical protein
VP0507	NO	branched chain amino acid transport system II carrier protein
VP0511	NO	ssDNA exonuclease RecJ
VP0512	NO	peptide chain release factor 2
VP0513	NO	lysyl-tRNA synthetase
VP0516	NO	aldo/keto reductase
VP0517	NO	hypothetical protein
VP0519	NO	hypothetical protein
VP0520	NO	dinucleoside polyphosphate hydrolase
VP0522	NO	hypothetical protein
VP0523	NO	prolipoprotein diacylglyceryl transferase
VP0525	NO	hypothetical protein
VP0527	NO	transcriptional activator NhaR
VP0528	NO	hypothetical protein
VP0529	NO	transcriptional activator HlyU
VP0531	NO	30S ribosomal protein S20
VP0532	NO	MviN protein
VP0533	NO	bifunctional riboflavin kinase/FMN adenylyltransferase
VP0534	NO	isoleucyl-tRNA synthetase
VP0536	NO	FKBP-type peptidylprolyl isomerase
VP0537	NO	4-hydroxy-3-methylbut-2-enyl diphosphate reductase
VP0538	NO	two-component response-regulatory protein YehT
VP0539	NO	hypothetical protein
VP0540	NO	carbon starvation protein A
VP0542	NO	hypothetical protein
VP0543	NO	N-acetylmuramic acid-6-phosphate etherase
VP0545	NO	beta-hexosaminidase
VP0546	NO	phospho-2-dehydro-3-deoxyheptonate aldolase
VP0547	NO	bifunctional chorismate mutase/prephenate dehydrogenase
VP0548	NO	ToxR-activated protein TagE

VP0549	NO	hypothetical protein
VP0551	NO	ABC transporter ATP-binding protein
VP0552	NO	soluble lytic murein transglycosylase
VP0553	NO	Trp operon repressor
VP0554	NO	NTPase
VP0555	NO	chorismate mutase/prephenate dehydratase
VP0556	NO	sigma-54 modulation protein
VP0558	NO	hypothetical protein
VP0560	NO	hypothetical protein
VP0561	NO	ClpB protein
VP0563	NO	hypothetical protein
VP0564	NO	penicillin-insensitive murein endopeptidase
VP0565	NO	hypothetical protein
VP0566	NO	ferredoxin
VP0569	NO	DNA-binding response regulator PhoB
VP0570	NO	phosphate regulon sensor protein
VP0571	NO	phosphate ABC transporter periplasmic phosphate-binding protein
VP0573	NO	polyphosphate kinase
VP0575	NO	phosphate ABC transporter permease
VP0576	NO	phosphate transporter ATP-binding protein
VP0577	NO	transcriptional regulator PhoU
VP0579	NO	copper homeostasis protein
VP0580	NO	anti-oxidant AhpCTSA family protein
VP0581	NO	LysR family transcriptional regulator
VP0582	NO	hypothetical protein
VP0583	NO	malate synthase
VP0584	NO	isocitrate lyase
VP0585	NO	acetoin utilization protein AcuB
VP0587	NO	S-adenosylmethionine:tRNA ribosyltransferase-isomerase
VP0588	NO	queuine tRNA-ribosyltransferase
VP0589	NO	preprotein translocase subunit YajC
VP0590	NO	preprotein translocase subunit SecD
VP0591	NO	preprotein translocase subunit SecF
VP0593	NO	inositol monophosphatase
VP0594	NO	RNA methyltransferase
VP0596	NO	cysteine desulfurase
VP0597	NO	scaffold protein
VP0598	NO	HesB family protein
VP0599	NO	co-chaperone HscB
VP0601	NO	ferredoxin
VP0602	NO	hypothetical protein
VP0604	NO	nucleoside diphosphate kinase
VP0605	NO	ribosomal RNA large subunit methyltransferase N
VP0607	NO	hypothetical protein
VP0608	NO	4-hydroxy-3-methylbut-2-en-1-yl diphosphate synthase
VP0610	NO	hypothetical protein
VP0611	NO	outer membrane protein assembly complex subunit YfgL
VP0613	NO	hypothetical protein
VP0614	NO	hypothetical protein
VP0615	NO	exodeoxyribonuclease VII large subunit
VP0616	NO	inosine 5'-monophosphate dehydrogenase
VP0617	NO	GMP synthase

VP0620	NO	hypothetical protein
VP0621	NO	nonspecific acid phosphatase
VP0623	NO	D-amino acid dehydrogenase small subunit
VP0624	NO	LysR family transcriptional regulator
VP0628	NO	hypothetical protein
VP0629	NO	homocysteine synthase
VP0630	NO	hypothetical protein
VP0632	NO	Na+/H+ antiporter
VP0636	NO	outer membrane protein A
VP0644	NO	SsrA-binding protein
VP0645	NO	hypothetical protein
VP0647	NO	small protein A
VP0648	NO	recombination and repair protein
VP0650	NO	inorganic polyphosphate/ATP-NAD kinase
VP0651	NO	heat shock protein GrpE
VP0652	NO	hypothetical protein
VP0653	NO	molecular chaperone DnaK
VP0654	NO	molecular chaperone DnaJ
VP0662	NO	hypothetical protein
VP0664	NO	hypothetical protein
VP0665	NO	transglycosylase
VP0669	NO	hypothetical protein
VP0670	NO	hypothetical protein
VP0671	NO	aminoacyl-histidine dipeptidase
VP0672	NO	hypothetical protein
VP0673	NO	xanthine-guanine phosphoribosyltransferase
VP0674	NO	fermentation/respiration switch protein
VP0676	NO	gamma-glutamyl kinase
VP0678	NO	transcriptional regulator NrdR
VP0679	NO	riboflavin-specific deaminase
VP0680	NO	riboflavin synthase subunit alpha
VP0681	NO	3,4-dihydroxy-2-butanone 4-phosphate synthase
VP0682	NO	6,7-dimethyl-8-ribityllumazine synthase
VP0683	NO	transcription antitermination protein NusB
VP0684	NO	thiamine monophosphate kinase
VP0686	NO	1-deoxy-D-xylulose-5-phosphate synthase
VP0687	NO	geranyltranstransferase
VP0688	NO	exodeoxyribonuclease VII small subunit
VP0689	NO	flagellar motor protein PomA
VP0690	NO	flagellar motor protein MotB
VP0691	NO	thiamine biosynthesis protein ThiI
VP0692	NO	DNA-binding transcriptional activator GcvA
VP0694	NO	hypothetical protein
VP0695	NO	RNA 2'-O-ribose methyltransferase
VP0697	NO	exonuclease IX
VP0698	NO	hypothetical protein
VP0699	NO	GGDEF family protein
VP0700	NO	hypothetical protein
VP0701	NO	7-cyano-7-deazaguanine reductase
VP0702	NO	SecY interacting protein Syd
VP0703	NO	hypothetical protein
VP0704	NO	DL-methionine transporter substrate-binding subunit

VP0706	NO	DL-methionine transporter ATP-binding subunit
VP0707	NO	hypothetical protein
VP0708	NO	D,D-heptose 1,7-bisphosphate phosphatase
VP0709	NO	trehalose repressor
VP0710	NO	PTS system trehalose(maltose)-specific transporter subunits IIBC
VP0711	NO	trehalose-6-phosphate hydrolase
VP0714	NO	hypothetical protein
VP0716	NO	lipoyl synthase
VP0717	NO	lipoate-protein ligase B
VP0718	NO	hypothetical protein
VP0719	NO	D-alanyl-D-alanine carboxypeptidase
VP0720	NO	rare lipoprotein A
VP0722	NO	penicillin-binding protein 2
VP0723	NO	rRNA large subunit methyltransferase
VP0724	NO	hypothetical protein
VP0725	NO	DNA polymerase III subunit delta
VP0726	NO	rare lipoprotein B
VP0727	NO	leucyl-tRNA synthetase
VP0728	NO	hypothetical protein
VP0729	NO	apolipoprotein N-acyltransferase
VP0730	NO	hemolysin
VP0731	NO	metalloprotease
VP0735	NO	2-octaprenyl-3-methyl-6-methoxy-1,4-benzoquinol hydroxylase
VP0737	NO	GTP-dependent nucleic acid-binding protein EngD
VP0738	NO	peptidyl-tRNA hydrolase
VP0739	NO	ribose-phosphate pyrophosphokinase
VP0742	NO	glutamyl-tRNA reductase
VP0743	NO	peptide chain release factor 1
VP0744	NO	HemK protein
VP0745	NO	hypothetical protein
VP0746	NO	hypothetical protein
VP0747	NO	2-dehydro-3-deoxyphosphooctonate aldolase
VP0748	NO	bifunctional UDP-sugar hydrolase/5'-nucleotidase periplasmic
VP0749	NO	hypothetical protein
VP0752	NO	hypothetical protein
VP0753	NO	hypothetical protein
VP0755	NO	N,N'-diacetylchitobiase
VP0756	NO	hypothetical protein
VP0757	NO	hypothetical protein
VP0759	NO	hypothetical protein
VP0763	NO	hypothetical protein
VP0768	NO	hypothetical protein
VP0770	NO	polar flagellar FlgN
VP0771	NO	polar flagellar FlgM
VP0774	NO	chemotaxis methyltransferase CheR
VP0781	NO	flagellar basal body rod protein FlgG
VP0783	NO	flagellar basal body P-ring biosynthesis protein FlgA
VP0786	NO	flagellar hook-associated protein FlgL
VP0793	NO	PTS system glucose-specific transporter subunit
VP0794	NO	phosphoenolpyruvate-protein phosphotransferase
VP0795	NO	phosphocarrier protein HPr
VP0796	NO	hypothetical protein

VP0797	NO	cysteine synthase A
VP0798	NO	sulfate transport protein CysZ
VP0802	NO	hypothetical protein
VP0804	NO	hypothetical protein
VP0806	NO	hypothetical protein
VP0807	NO	hypothetical protein
VP0808	NO	short chain dehydrogenase
VP0809	NO	sugar nucleotide epimerase
VP0810	NO	PTS system mannose-specific, factor IIC
VP0812	NO	hypothetical protein
VP0814	NO	hypothetical protein
VP0815	NO	hypothetical protein
VP0816	NO	phosphodiesterase
VP0817	NO	selenoprotein W-like protein
VP0818	NO	hypothetical protein
VP0819	NO	ToxS protein
VP0820	NO	ToxR protein
VP0821	NO	heat shock protein 90
VP0822	NO	adenylate kinase
VP0823	NO	ferrochelatase
VP0824	NO	permease
VP0825	NO	transcriptional activator RfaH
VP0826	NO	asparagine synthetase B
VP0827	NO	glutathione-regulated potassium-efflux system protein KefB
VP0828	NO	N-acetylglucosamine repressor
VP0829	NO	N-acetylglucosamine-6-phosphate deacetylase
VP0831	NO	PTS system N-acetylglucosamine-specific transporter subunit IIABC
VP0832	NO	glutaminyl-tRNA synthetase
VP0833	NO	ferric uptake regulator
VP0834	NO	hypothetical protein
VP0835	NO	flavodoxin FldA
VP0836	NO	hypothetical protein
VP0837	NO	esterase/lipase YbfF
VP0840	NO	hypothetical protein
VP0841	NO	hypothetical protein
VP0842	NO	type II citrate synthase
VP0845	NO	succinate dehydrogenase flavoprotein subunit
VP0846	NO	succinate dehydrogenase iron-sulfur subunit
VP0847	NO	2-oxoglutarate dehydrogenase E1
VP0850	NO	succinyl-CoA synthetase subunit alpha
VP0853	NO	zinc ABC transporter periplasmic zinc-binding protein
VP0854	NO	AraC family transcriptional regulator
VP0857	NO	ferrous iron transport protein A
VP0861	NO	arginyl-tRNA synthetase
VP0862	NO	hypothetical protein
VP0865	NO	ATP-dependent helicase DinG
VP0866	NO	hypothetical protein
VP0867	NO	hypothetical protein
VP0868	NO	outer membrane lipoprotein Slp
VP0869	NO	hypothetical protein
VP0870	NO	long-chain-fatty-acid--CoA ligase
VP0871	NO	ribonuclease D

VP0872	NO	cell division topological specificity factor MinE
VP0873	NO	septum site-determining protein MinD
VP0874	NO	septum formation inhibitor
VP0877	NO	LysR family transcriptional regulator
VP0878	NO	NupC family protein
VP0879	NO	bifunctional 5,10-methylene-tetrahydrofolate dehydrogenase/ 5,10-methylene-tetrahydrofolate cyclohydrolase
VP0881	NO	3-oxoacyl-ACP synthase
VP0882	NO	3-ketoacyl-ACP reductase
VP0884	NO	3-oxoacyl-ACP synthase
VP0885	NO	lipoprotein
VP0886	NO	hypothetical protein
VP0888	NO	hypothetical protein
VP0890	NO	hypothetical protein
VP0894	NO	acyl carrier protein
VP0895	NO	acyl carrier protein
VP0896	NO	phospholipid biosynthesis acyltransferase
VP0898	NO	lipoprotein
VP0899	NO	O-methyltransferase
VP0900	NO	oxidoreductase
VP0902	NO	hemolysin
VP0903	NO	RhlE protein
VP0904	NO	KtrA protein
VP0907	NO	hypothetical protein
VP0910	NO	C4-dicarboxylate-binding periplasmic protein
VP0911	NO	C4-dicarboxylate transport protein DctQ
VP0912	NO	C4-dicarboxylate transport protein
VP0913	NO	hypothetical protein
VP0915	NO	C4-dicarboxylate transport sensor protein
VP0916	NO	trigger factor
VP0917	NO	ATP-dependent Clp protease proteolytic subunit
VP0918	NO	ATP-dependent protease ATP-binding subunit ClpX
VP0919	NO	ATP-dependent protease LA
VP0920	NO	DNA-binding protein HU-beta
VP0921	NO	peptidyl-prolyl cis-trans isomerse D
VP0922	NO	hypothetical protein
VP0923	NO	hypothetical protein
VP0925	NO	deoxyguanosinetriphosphate triphosphohydrolase-like protein
VP0927	NO	aminotransferase
VP0928	NO	menaquinone-specific isochorismate synthase
VP0931	NO	naphthoate synthase
VP0933	NO	O-succinylbenzoic acid--CoA ligase
VP0936	NO	hypothetical protein
VP0937	NO	hypothetical protein
VP0939	NO	hypothetical protein
VP0940	NO	hypothetical protein
VP0941	NO	multidrug resistance protein
VP0942	NO	fimbrial protein
VP0947	NO	AsnC family transcriptional regulator
VP0949	NO	acyl-CoA thioesterase
VP0950	NO	lipoprotein-like protein
VP0951	NO	methylated-DNA-protein-cysteine methyltransferase-like protein
VP0953	NO	hypothetical protein

VP0954	NO	RNA methyltransferase
VP0955	NO	ferredoxin
VP0956	NO	hypothetical protein
VP0958	NO	inosine monophosphate dehydrogenase-like protein
VP0959	NO	zinc/cadmium/mercury/lead-transporting ATPase
VP0960	NO	uridine phosphorylase
VP0962	NO	hypothetical protein
VP0963	NO	methyl-accepting chemotaxis protein
VP0964	NO	Hit family protein
VP0965	NO	hypothetical protein
VP0966	NO	hypothetical protein
VP0967	NO	hypothetical protein
VP0969	NO	hypothetical protein
VP0971	NO	NADH dehydrogenase
VP0973	NO	ribosomal-protein-serine acetyltransferase
VP0975	NO	transcription-repair coupling factor
VP0976	NO	hypothetical protein
VP0977	NO	hypothetical protein
VP0979	NO	outer membrane-specific lipoprotein transporter subunit LolE
VP0980	NO	hypothetical protein
VP0982	NO	lipid transporter ATP-binding/permease
VP0983	NO	tetraacyldisaccharide 4'-kinase
VP0984	NO	3-deoxy-manno-octulosonate cytidylyltransferase
VP0986	NO	hypothetical protein
VP0988	NO	hypothetical protein
VP0990	NO	hypothetical protein
VP0991	NO	hypothetical protein
VP0992	NO	pyruvate formate lyase-activating enzyme 1
VP0993	NO	hypothetical protein
VP0994	NO	formate acetyltransferase
VP0995	NO	hypothetical protein
VP0998	NO	amino acid ABC transporter ATP-binding protein
VP0999	NO	amino acid ABC transporter substrate-binding protein
VP1000	NO	amino acid ABC transporter permease
VP1003	NO	exonuclease III
VP1004	NO	hypothetical protein
VP1005	NO	primosomal replication protein N''
VP1006	NO	hypothetical protein
VP1007	NO	ATP-dependent DNA helicase DinG
VP1011	NO	isocitrate dehydrogenase
VP1013	NO	ATP-dependent Clp protease adaptor protein ClpS
VP1016	NO	translation initiation factor IF-1
VP1017	NO	arginyl-tRNA-protein transferase
VP1018	NO	leucyl/phenylalanyl-tRNA--protein transferase
VP1019	NO	outer membrane protein
VP1020	NO	3-phosphoshikimate 1-carboxyvinyltransferase
VP1021	NO	hypothetical protein
VP1022	NO	DNA topoisomerase I
VP1025	NO	beta-ketoadipate enol-lactone hydrolase
VP1026	NO	hypothetical protein
VP1027	NO	hypothetical protein
VP1028	NO	hypothetical protein

VP1030	NO	LacI family transcription regulator
VP1031	NO	chaperone protein TorD
VP1032	NO	DNA-binding transcriptional regulator TorR
VP1033	NO	hypothetical protein
VP1034	NO	metallothionein SmtA
VP1035	NO	condesin subunit F
VP1037	NO	cell division protein MukB
VP1038	NO	hypothetical protein
VP1040	NO	hypothetical protein
VP1041	NO	gonadoliberin III-like protein
VP1043	NO	hypothetical protein
VP1044	NO	hypothetical protein
VP1045	NO	hypothetical protein
VP1047	NO	aspartyl-tRNA synthetase
VP1048	NO	Holliday junction resolvase
VP1051	NO	Holliday junction DNA helicase RuvA
VP1052	NO	Holliday junction DNA helicase RuvB
VP1053	NO	cytochrome d ubiquinol oxidase subunit I
VP1054	NO	cytochrome d ubiquinol oxidase subunit II
VP1057	NO	TolQ protein
VP1060	NO	translocation protein TolB
VP1061	NO	peptidoglycan-associated lipoprotein
VP1062	NO	hypothetical protein
VP1063	NO	quinolinate synthetase
VP1100	NO	hypothetical protein
VP1101	NO	transcriptional regulator CysB
VP1106	NO	outer-membrane lipoprotein carrier protein
VP1107	NO	recombination factor protein RarA
VP1112	NO	adenosylmethionine-8-amino-7-oxononanoate aminotransferase
VP1114	NO	8-amino-7-oxononanoate synthase
VP1115	NO	biotin synthesis protein BioC
VP1117	NO	hypothetical protein
VP1119	NO	transcriptional regulator
VP1120	NO	short chain dehydrogenase
VP1121	NO	hypothetical protein
VP1123	NO	cyclopropane-fatty-acyl-phospholipid synthase
VP1125	NO	hypothetical protein
VP1126	NO	hypothetical protein
VP1128	NO	adenylosuccinate lyase
VP1129	NO	hypothetical protein
VP1130	NO	tRNA-specific 2-thiouridylase MnmA
VP1131	NO	hypothetical protein
VP1133	NO	DNA-binding protein H-NS
VP1134	NO	hypothetical protein
VP1135	NO	hypothetical protein
VP1136	NO	transcription regulator TxR
VP1137	NO	ATP phosphoribosyltransferase
VP1138	NO	histidinol dehydrogenase
VP1139	NO	histidinol-phosphate aminotransferase
VP1140	NO	imidazole glycerol-phosphate dehydratase/histidinol phosphatase
VP1141	NO	imidazole glycerol phosphate synthase subunit HisH
VP1143	NO	imidazole glycerol phosphate synthase subunit HisF

VP1144	NO	bifunctional phosphoribosyl-AMP cyclohydrolase/phosphoribosyl-ATP pyrophosphatase protein
VP1145	NO	hypothetical protein
VP1147	NO	hypothetical protein
VP1148	NO	UDP-2,3-diacylglucosamine hydrolase
VP1149	NO	peptidyl-prolyl cis-trans isomerase B
VP1150	NO	cysteinyl-tRNA synthetase
VP1151	NO	thymidine kinase
VP1152	NO	hypothetical protein
VP1153	NO	hypothetical protein
VP1156	NO	DNA-binding protein inhibitor Id-2-like protein
VP1157	NO	formate transporter 1
VP1158	NO	hypothetical protein
VP1160	NO	NapE protein
VP1161	NO	cytochrome c-type protein TorC
VP1162	NO	trimethylamine-N-oxide reductase
VP1163	NO	alpha-1,6-galactosidase
VP1164	NO	hypothetical protein
VP1165	NO	manganese-dependent inorganic pyrophosphatase
VP1166	NO	hypothetical protein
VP1167	NO	peptide ABC transporter ATP-binding protein
VP1168	NO	peptide ABC transporter ATP-binding protein
VP1170	NO	peptide ABC transporter permease
VP1171	NO	peptide ABC transporter periplasmic peptide-binding protein
VP1172	NO	psp operon transcriptional activator
VP1173	NO	phage shock protein A
VP1175	NO	phage shock protein C
VP1176	NO	multidrug resistance protein
VP1177	NO	periplasmic linker protein
VP1179	NO	DNA-3-methyladenine glycosidase I
VP1181	NO	lactonizing lipase
VP1182	NO	cystathionine beta-lyase
VP1183	NO	cardiolipin synthetase
VP1185	NO	chemotaxis transducer
VP1186	NO	pseudouridine synthase
VP1187	NO	hypothetical protein
VP1188	NO	ferredoxin oxidoreductase protein
VP1189	NO	hydroxylamine reductase
VP1190	NO	anaerobic nitric oxide reductase transcription regulator
VP1191	NO	hypothetical protein
VP1192	NO	outer membrane lipoprotein Pcp
VP1195	NO	ABC transporter permease
VP1196	NO	ABC transporter solute-binding protein
VP1197	NO	hypothetical protein
VP1200	NO	thiosulfate sulfurtransferase
VP1203	NO	heat shock protein HslJ
VP1204	NO	hypothetical protein
VP1210	NO	50S ribosomal protein L25
VP1213	NO	hypothetical protein
VP1214	NO	helicase-like protein
VP1215	NO	ribosomal small subunit pseudouridine synthase A
VP1218	NO	outer membrane protein
VP1219	NO	deca-heme c-type cytochrome

VP1221	NO	cytochrome subunit of sulfide dehydrogenase
VP1223	NO	hypothetical protein
VP1226	NO	hypothetical protein
VP1227	NO	hypothetical protein
VP1228	NO	pH-dependent sodium/proton antiporter
VP1229	NO	Na+/H+-antiporter protein
VP1230	YES	acyl-CoA dehydrogenase
VP1231	YES	trans-2-enoyl-CoA reductase
VP1232	YES	hypothetical protein
VP1233	YES	glutaredoxin
VP1235	YES	iron-containing alcohol dehydrogenase
VP1236	YES	DNA-binding transcriptional regulator HexR
VP1237	YES	glutamate decarboxylase
VP1238	NO	hypothetical protein
VP1239	NO	hypothetical protein
VP1240	NO	hypothetical protein
VP1241	NO	hypothetical protein
VP1243	NO	hypothetical protein
VP1244	NO	response regulator
VP1245	NO	response regulator
VP1247	NO	phosphoserine aminotransferase
VP1251	NO	thioredoxin reductase
VP1253	NO	NifS-like protein
VP1261	NO	hypothetical protein
VP1265	NO	hypothetical protein
VP1267	NO	lipoprotein
VP1274	NO	urocanate hydratase
VP1276	NO	imidazolonepropionase
VP1280	NO	threonyl-tRNA synthetase
VP1281	NO	50S ribosomal protein L35
VP1282	NO	50S ribosomal protein L20
VP1284	NO	hypothetical protein
VP1287	NO	hypothetical protein
VP1290	NO	phenylalanyl-tRNA synthetase subunit alpha
VP1293	NO	hypothetical protein
VP1294	NO	integration host factor subunit alpha
VP1296	NO	thiopurine S-methyltransferase
VP1297	NO	phosphoribosylglycinamide formyltransferase 2
VP1299	NO	hypothetical protein
VP1300	NO	hypothetical protein
VP1302	NO	sodium/dicarboxylate symporter
VP1306	NO	adenosylcobinamide kinase
VP1311	NO	vtamin B12-transporter permease
VP1313	NO	hypothetical protein
VP1315	NO	multidrug resistance protein
VP1316	NO	LysR family transcriptional regulator
VP1324	NO	SAM-dependent methyltransferase
VP1327	NO	hypothetical protein
VP1330	NO	hydroxyproline-2-epimerase
VP1331	NO	D-amino acid dehydrogenase, small subunit
VP1332	NO	binding protein component of ABC transporter
VP1333	NO	ornithine cyclodeaminase

VP1335	NO	dihydrodipicolinate synthetase
VP1342	NO	aminopeptidase
VP1343	NO	oligopeptide ABC transporter ATP-binding protein
VP1347	NO	oligopeptide ABC transporter periplasmic oligopeptide-binding protein
VP1348	NO	M20A family peptidase
VP1350	NO	oxidoreductase
VP1352	NO	glutathione S-transferase
VP1354	NO	hypothetical protein
VP1374	NO	transporter
VP1377	NO	hypothetical protein
VP1378	NO	HD-GYP domain-containing protein
VP1379	NO	homoserine/homoserine lactone efflux protein
VP1381	NO	pyruvate formate lyase activating enzyme
VP1422	NO	SM-20-like protein
VP1423	NO	hypothetical protein
VP1426	NO	tyrosine-specific transport protein
VP1427	NO	hypothetical protein
VP1430	NO	hypothetical protein
VP1431	NO	ATP-binding protein of a transport system
VP1434	NO	V10 pilin
VP1436	NO	hypothetical protein
VP1437	NO	hypothetical protein
VP1438	NO	molecular chaperone DnaK
VP1442	NO	hypothetical protein
VP1443	NO	hypothetical protein
VP1445	NO	hypothetical protein
VP1447	NO	anaerobic dimethyl sulfoxide reductase subunit A
VP1448	NO	anaerobic dimethyl sulfoxide reductase subunit B
VP1449	NO	anaerobic dimethyl sulfoxide reductase subunit C
VP1450	NO	component of anaerobic dehydrogenase
VP1452	NO	azoreductase
VP1454	NO	hypothetical protein
VP1455	NO	hypothetical protein
VP1457	NO	hypothetical protein
VP1458	NO	hypothetical protein
VP1459	NO	intercellular adhesion protein A
VP1460	NO	hypothetical protein
VP1463	NO	glycosyltransferase
VP1464	NO	ExoQ family protein
VP1465	NO	lipopolysaccharide biosynthesis protein
VP1469	NO	LuxO repressor protein
VP1472	NO	phosphorelay protein
VP1473	NO	capsular polysaccharide biosynthesis
VP1474	NO	capsule transport protein OtnA
VP1475	NO	hypothetical protein
VP1476	NO	anti-sigma F factor antagonist
VP1479	NO	multidrug efflux protein
VP1481	NO	hypothetical protein
VP1482	NO	response regulator
VP1484	NO	hypothetical protein
VP1485	NO	lipoprotein NlpC
VP1486	NO	methyl-accepting chemotaxis protein

VP1494	NO	N-acetyl-D-glucosamine kinase
VP1495	NO	hypothetical protein
VP1501	NO	hypothetical protein
VP1503	NO	sensor histidine kinase
VP1504	NO	ABC transporter ATP-binding protein
VP1506	NO	formate dehydrogenase accessory protein
VP1511	NO	formate dehydrogenase-specific chaperone
VP1512	NO	hypothetical protein
VP1513	NO	formate dehydrogenase large subunit
VP1514	NO	formate dehydrogenase, iron-sulfur subunit
VP1515	NO	formate dehydrogenase, cytochrome b556 subunit
VP1524	NO	NAD-dependent deacetylase
VP1525	NO	spermidine/putrescine ABC transporter periplasmic spermidine/putrescine-binding protein
VP1526	NO	spermidine/putrescine ABC transporter periplasmic spermidine/putrescine-binding protein
VP1527	NO	spermidine/putrescine ABC transporter membrane protein
VP1529	NO	putrescine/spermidine ABC transporter ATPase protein
VP1531	NO	Bax protein
VP1532	NO	hypothetical protein
VP1533	NO	C32 tRNA thiolase
VP1535	NO	universal stress protein UspE
VP1536	NO	fumarate/nitrate reduction transcriptional regulator
VP1537	NO	hypothetical protein
VP1538	NO	FixS-like protein
VP1539	NO	cation transporter E1-E2 family ATPase
VP1540	NO	hypothetical protein
VP1541	NO	cytochrome c oxidase subunit CcoP
VP1542	NO	cytochrome c oxidase subunit CcoQ
VP1544	NO	cbb3-type cytochrome c oxidase subunit I
VP1588	NO	hypothetical protein
VP1593	NO	ribosome modulation factor
VP1598	NO	hypothetical protein
VP1599	NO	hypothetical protein
VP1603	NO	hypothetical protein
VP1608	NO	hypothetical protein
VP1609	NO	ABC transporter ATP-binding protein
VP1611	NO	hypothetical protein
VP1612	NO	rRNA (cytosine-C(5)-)-methyltransferase RsmF
VP1613	NO	hypothetical protein
VP1614	NO	hypothetical protein
VP1615	NO	hypothetical protein
VP1616	NO	phospho-2-dehydro-3-deoxyheptonate aldolase
VP1617	NO	nucleotide-binding protein
VP1620	NO	amino acid ABC transporter substrate-binding protein
VP1621	NO	amino acid ABC transporter permease
VP1622	NO	amino acid ABC transporter permease
VP1625	NO	hypothetical protein
VP1626	NO	sulfite reductase, gamma subunit-like protein
VP1627	NO	acylphosphatase
VP1629	NO	SAM-dependent methyltransferase
VP1656	NO	translocator protein PopD
VP1658	NO	low calcium response locus protein H
VP1662	NO	low calcium response protein

VP1665	YES	type III secretion protein
VP1669	YES	type III secretion protein YscO
VP1672	YES	type III secretion system protein
VP1673	YES	translocation protein in type III secretion
VP1674	YES	translocation protein in type III secretion
VP1683	YES	hypothetical protein
VP1684	YES	hypothetical protein
VP1687	YES	type III chaperone
VP1690	YES	type III secretion lipoprotein
VP1691	YES	type III export protein
VP1693	YES	type III secretion protein
VP1694	YES	type III export protein YscF
VP1695	YES	type III export protein PscD
VP1696	YES	type III secretion protein YscC
VP1698	NO	hypothetical protein
VP1699	NO	transcriptional regulator ExsA
VP1702	NO	hypothetical protein
VP1705	NO	hypothetical protein
VP1706	NO	hypothetical protein
VP1708	NO	6-phosphogluconate dehydrogenase
VP1709	NO	6-phosphogluconolactonase
VP1710	NO	glucose-6-phosphate 1-dehydrogenase
VP1711	NO	response regulator
VP1712	NO	sensor kinase CitA
VP1713	NO	hypothetical protein
VP1717	NO	hypothetical protein
VP1719	NO	aspartate kinase
VP1720	NO	L-ectoine synthase
VP1724	NO	DNA polymerase III subunit epsilon
VP1725	NO	hypothetical protein
VP1729	NO	thermostable hemolysin delta-VPH
VP1730	NO	long-chain-fatty-acid-CoA ligase
VP1731	NO	hypothetical protein
VP1732	NO	short chain dehydrogenase
VP1733	NO	hypothetical protein
VP1736	NO	hypothetical protein
VP1737	NO	hypothetical protein
VP1738	NO	hypothetical protein
VP1740	NO	hypothetical protein
VP1741	NO	sodium/alanine symporter
VP1743	NO	hypothetical protein
VP1745	NO	lipid A biosynthesis lauroyl acyltransferase
VP1751	NO	homoserine O-succinyltransferase
VP1752	NO	fimbrial biogenesis and twitching motility protein
VP1753	NO	alkylphosphonate ABC transporter
VP1757	NO	hypothetical protein
VP1758	NO	galactoside O-acetyltransferase
VP1760	NO	adenylate cyclase
VP1763	NO	MarR family transcriptional regulator
VP1764	NO	transmembrane protein
VP1768	NO	hypothetical protein
VP1866	NO	hypothetical protein

VP1868	NO	ribosomal-protein-alanine acetyltransferase
VP1869	NO	transcriptional regulator TyrR
VP1871	NO	hypothetical protein
VP1872	NO	hypothetical protein
VP1873	NO	fumarate hydratase, class I
VP1876	NO	hypothetical protein
VP1877	NO	guanylate cyclase-like protein
VP1878	NO	MutT/nudix family protein
VP1879	NO	serine transporter
VP1880	NO	L-serine dehydratase 1
VP1881	NO	hypothetical protein
VP1882	NO	hypothetical protein
VP1892	NO	methyl-accepting chemotaxis protein
VP1893	NO	asparaginyl-tRNA synthetase
VP1897	NO	hypothetical protein
VP1900	NO	aromatic amino acid aminotransferase
VP1901	NO	outer membrane protein Ail and OmpX
VP1903	NO	DNA polymerase III subunit epsilon
VP1904	NO	methyl-accepting chemotaxis protein
VP1905	NO	BCCT family transporter
VP1906	NO	MarR family transcriptional regulator
VP1908	NO	sensor histidine kinase
VP1909	NO	hypothetical protein
VP1911	NO	ABC transporter substrate-binding protein
VP1913	NO	hypothetical protein
VP1914	NO	glyoxylase
VP1915	NO	hypothetical protein
VP1916	NO	amidase
VP1919	NO	hypothetical protein
VP1921	NO	GTP cyclohydrolase II
VP1922	NO	hypothetical protein
VP1926	NO	formate dependent nitrate reductase NrfD
VP1927	NO	nitrite reductase Fe-S protein NrfC
VP1928	NO	cytochrome c nitrite reductase pentaheme subunit
VP1929	NO	cytochrome c552
VP1931	NO	YfrE protein
VP1933	NO	3-demethylubiquinone-9 3-methyltransferase
VP1934	NO	ribonucleotide-diphosphate reductase subunit alpha
VP1935	NO	ribonucleotide-diphosphate reductase subunit beta
VP1936	NO	iron-sulfur cluster-binding protein
VP1939	NO	hypothetical protein
VP1940	NO	carboxynorspermidine decarboxylase
VP1943	NO	CDP-diacylglycerol--glycerol-3-phosphate 3-phosphatidyltransferase
VP1944	NO	excinuclease ABC subunit C
VP1947	NO	hypothetical protein
VP1948	NO	elongation factor P
VP1949	NO	dsDNA-mimic protein
VP1951	NO	pseudouridine synthase
VP1952	NO	hypothetical protein
VP1953	NO	translation factor
VP1954	NO	metal-dependent phosphoesterase YciV
VP1955	NO	Trp operon leader peptide

VP1956	NO	anthranilate synthase component I
VP1957	NO	anthranilate synthase component II
VP1958	NO	anthranilate phosphoribosyltransferase
VP1959	NO	bifunctional indole-3-glycerol phosphate synthase/phosphoribosylanthranilate isomerase
VP1960	NO	tryptophan synthase subunit beta
VP1961	NO	tryptophan synthase subunit alpha
VP1962	NO	transcriptional regulator
VP1969	NO	hypothetical protein
VP1970	NO	intracellular septation protein A
VP1971	NO	acyl-CoA hydrolase
VP1978	NO	hypothetical protein
VP1982	NO	hypothetical protein
VP1983	NO	two-component response regulator
VP1987	NO	hypothetical protein
VP1988	NO	LysR family transcriptional regulator
VP1989	NO	secretion protein
VP1991	NO	5-methyltetrahydropteroyltriglutamate-- homocysteine S-methyltransferase
VP1992	NO	hypothetical protein
VP1996	NO	hypothetical protein
VP1997	NO	hypothetical protein
VP1998	NO	outer membrane protein TolC
VP2006	NO	suppressor for copper-sensitivity B
VP2009	NO	tetrathionate reductase complex: response regulator
VP2011	NO	tetrathionate reductase subunit B
VP2013	NO	tetrathionate reductase complex subunit C
VP2014	NO	tetrathionate reductase subunit A
VP2015	NO	cytochrome c
VP2018	NO	paraquat-inducible protein B
VP2019	NO	hypothetical protein
VP2020	NO	hypothetical protein
VP2021	NO	hypothetical protein
VP2022	NO	glycosyl transferase family protein
VP2026	NO	orotidine 5'-phosphate decarboxylase
VP2027	NO	tetratricopeptide repeat protein
VP2028	NO	hypothetical protein
VP2029	NO	integration host factor subunit beta
VP2031	NO	cytidylate kinase
VP2032	NO	periplasmic protease
VP2033	YES	short chain dehydrogenase
VP2034	YES	hypothetical protein
VP2035	YES	hypothetical protein
VP2036	YES	DNA polymerase III alpha chain
VP2037	YES	chemotaxis protein CheV
VP2038	YES	ROK family transcriptional regulator
VP2039	YES	pyruvate kinase II
VP2044	NO	hypothetical protein
VP2048	NO	DNA polymerase III subunit delta'
VP2049	NO	thymidylate kinase
VP2050	NO	hypothetical protein
VP2051	NO	4-amino-4-deoxychorismate lyase
VP2052	NO	3-oxoacyl-ACP synthase
VP2053	NO	acyl carrier protein

VP2054	NO	3-ketoacyl-ACP reductase
VP2055	NO	ACP S-malonyltransferase
VP2056	NO	3-oxoacyl-ACP synthase
VP2057	NO	glycerol-3-phosphate acyltransferase PlsX
VP2058	NO	50S ribosomal protein L32
VP2059	NO	hypothetical protein
VP2061	NO	23S rRNA ribosomal pseudouridine synthase
VP2063	NO	sulfate permease
VP2064	NO	phosphotyrosine protein phosphatase
VP2065	NO	cob(I)yrinic acid a,c-diamide adenosyltransferase
VP2066	NO	AsmA protein
VP2068	NO	Mrp protein
VP2069	NO	methionyl-tRNA synthetase
VP2071	NO	fatty acid metabolism regulator
VP2072	NO	sodium/proton antiporter
VP2074	NO	preprotein translocase SecA subunit-like protein
VP2078	NO	ABC transporter ATP-binding protein
VP2079	NO	ABC transporter permease
VP2080	NO	ABC transporter substrate-binding protein
VP2081	NO	hypothetical protein
VP2082	NO	acetate kinase
VP2083	NO	phosphate acetyltransferase
VP2084	NO	hypothetical protein
VP2086	NO	glutathione S-transferase
VP2087	NO	oligopeptide ABC transporter ATP-binding protein
VP2089	NO	oligopeptide ABC transporter permease
VP2090	NO	oligopeptide transporter permease
VP2091	NO	oligopeptide ABC transporter periplasmic oligopeptide-binding protein
VP2092	NO	molybdenum cofactor biosynthesis protein E
VP2093	NO	molybdopterin synthase small subunit
VP2094	NO	molybdenum cofactor biosynthesis protein MoaC
VP2095	NO	molybdenum cofactor biosynthesis protein B
VP2096	NO	molybdenum cofactor biosynthesis protein A
VP2097	NO	hypothetical protein
VP2100	NO	excinuclease ABC subunit B
VP2102	NO	Na(+)-translocating NADH-quinone reductase subunit E
VP2103	NO	electron transport complex protein RnfB
VP2105	NO	electron transport complex protein RnfD
VP2106	NO	electron transport complex protein RnfG
VP2109	NO	lactoylglutathione lyase
VP2110	NO	hypothetical protein
VP2111	NO	sodium-type flagellar protein MotY
VP2112	NO	MotY protein
VP2113	NO	hypothetical protein
VP2114	NO	ribonuclease T
VP2115	NO	hypothetical protein
VP2116	NO	hypothetical protein
VP2117	NO	glutaredoxin protein
VP2118	NO	manganese superoxide dismutase Mn-SOD
VP2119	NO	hypothetical protein
VP2120	NO	short chain dehydrogenase
VP2121	NO	bifunctional acetaldehyde-CoA/alcohol dehydrogenase

VP2122	NO	hypothetical protein
VP2123	NO	potassium channel
VP2124	NO	aspartate-semialdehyde dehydrogenase
VP2125	NO	Na+/H+ antiporter
VP2126	NO	hypothetical protein
VP2127	NO	hypothetical protein
VP2128	NO	nucleoid-associated protein NdpA
VP2129	NO	hypothetical protein
VP2130	NO	hypothetical protein
VP2147	NO	hypothetical protein
VP2149	NO	DNA topoisomerase III
VP2151	NO	2,4-dienoyl-CoA reductase
VP2152	NO	protease IV
VP2153	NO	cytoplasmic asparaginase I
VP2154	NO	hypothetical protein
VP2156	NO	methionine sulfoxide reductase B
VP2157	NO	glyceraldehyde-3-phosphate dehydrogenase
VP2158	NO	hypothetical protein
VP2161	NO	hypothetical protein
VP2162	NO	hypothetical protein
VP2163	NO	alkaline phosphatase
VP2165	NO	hypothetical protein
VP2166	NO	lactoylglutathione lyase
VP2167	NO	hypothetical protein
VP2168	NO	hypothetical protein
VP2170	NO	transglycosylase associated gene
VP2171	NO	proteinase inhibitor
VP2173	NO	hypothetical protein
VP2176	NO	aquaporin Z
VP2177	NO	recombination protein RecR
VP2178	NO	hypothetical protein
VP2180	NO	adenine phosphoribosyltransferase
VP2181	NO	hypothetical protein
VP2182	NO	hypothetical protein
VP2184	NO	LysR family transcriptional regulator
VP2185	NO	amidophosphoribosyltransferase
VP2186	NO	bacteriocin production protein
VP2189	NO	acetyl-CoA carboxylase subunit beta
VP2190	NO	tRNA pseudouridine synthase A
VP2192	NO	aspartate-semialdehyde dehydrogenase
VP2193	NO	erythronate-4-phosphate dehydrogenase
VP2194	NO	3-oxoacyl-ACP synthase
VP2195	NO	5-methylaminomethyl-2-thiouridine methyltransferase
VP2197	NO	hypothetical protein
VP2198	NO	hypothetical protein
VP2200	NO	hypothetical protein
VP2201	NO	hypothetical protein
VP2203	NO	N5-glutamine S-adenosyl-L-methionine-dependent methyltransferase
VP2204	NO	hypothetical protein
VP2207	NO	CG2 omega domain-containing protein
VP2208	NO	multifunctional fatty acid oxidation complex subunit alpha
VP2209	NO	3-ketoacyl-CoA thiolase

VP2211	NO	hypothetical protein
VP2214	NO	VacJ lipoprotein
VP2215	NO	cytochrome c-type biogenesis protein
VP2216	NO	cytochrome c-type biogenesis protein
VP2217	NO	thiol:disulfide interchange protein DsbE
VP2219	NO	cytochrome c-type biogenesis protein CcmE
VP2220	NO	heme exporter protein D
VP2222	NO	heme exporter protein B
VP2223	NO	cytochrome c biogenesis protein CcmA
VP2224	NO	hypothetical protein
VP2225	NO	chemotaxis protein CheW
VP2230	NO	chemotaxis protein CheZ
VP2231	NO	chemotaxis protein CheY
VP2233	NO	flagellar biosynthesis protein FlhG
VP2234	NO	flagellar biosynthesis regulator FlhF
VP2235	NO	flagellar biosynthesis protein FlhA
VP2236	NO	flagellar biosynthesis protein FlhB
VP2237	NO	flagellar biosynthesis protein FliR
VP2238	NO	flagellar biosynthesis protein FliQ
VP2239	NO	flagellar biosynthesis protein FliP
VP2241	NO	flagellar motor switch protein
VP2242	NO	flagellar motor switch protein FliM
VP2243	NO	flagellar basal body protein FliL
VP2245	NO	flagellar biosynthesis chaperone
VP2250	NO	flagellar hook-basal body protein FliE
VP2255	NO	polar flagellar rod protein FlaI
VP2257	NO	flagellar protein FlaG
VP2261	NO	flagellin
VP2262	NO	TyrA protein
VP2263	NO	hypothetical protein
VP2264	NO	hypothetical protein
VP2265	NO	outer membrane lipoprotein
VP2266	NO	hypothetical protein
VP2269	NO	succinyl-diaminopimelate desuccinylase
VP2270	NO	D,D-carboxypeptidase-like protein
VP2271	NO	hypothetical protein
VP2272	NO	lipoprotein
VP2273	NO	dihydrodipicolinate synthase
VP2274	NO	glycine cleavage system transcriptional repressor
VP2276	NO	permease PerM
VP2277	NO	hypothetical protein
VP2278	NO	hypothetical protein
VP2279	NO	arsenate reductase
VP2280	NO	Trp repressor-binding protein
VP2281	NO	hypothetical protein
VP2283	NO	uracil permease
VP2284	NO	uracil phosphoribosyltransferase
VP2285	NO	phosphoribosylaminoimidazole synthetase
VP2286	NO	phosphoribosylglycinamide formyltransferase
VP2287	NO	amidotransferase
VP2288	NO	phosphoheptose isomerase
VP2289	NO	acyl-CoA dehydrogenase

VP2290	NO	sulfate permease
VP2292	NO	DNA polymerase III subunit epsilon
VP2293	NO	ribonuclease H
VP2295	NO	hydroxyacylglutathione hydrolase GloB
VP2296	NO	membrane-bound lytic murein transglycosylase D
VP2297	NO	hypothetical protein
VP2298	NO	hypothetical protein
VP2299	NO	nitrogen regulatory protein P-II
VP2300	NO	cytochrome c554
VP2301	NO	cell cycle protein MesJ
VP2302	NO	acetyl-CoA carboxylase carboxyltransferase subunit alpha
VP2303	NO	DNA polymerase III subunit alpha
VP2304	NO	ribonuclease HII
VP2305	NO	lipid-A-disaccharide synthase
VP2306	NO	UDP-N-acetylglucosamine acyltransferase
VP2307	NO	(3R)-hydroxymyristoyl-ACP dehydratase
VP2308	NO	UDP-3-O-[3-hydroxymyristoyl] glucosamine N-acyltransferase
VP2310	NO	outer membrane protein assembly factor YaeT
VP2311	NO	membrane-associated Zn-dependent protease
VP2312	NO	1-deoxy-D-xylulose 5-phosphate reductoisomerase
VP2313	NO	phosphatidate cytidylyltransferase
VP2314	NO	undecaprenyl diphosphate synthase
VP2315	NO	ribosome recycling factor
VP2316	NO	uridylate kinase
VP2317	NO	elongation factor Ts
VP2318	NO	30S ribosomal protein S2
VP2319	NO	methionine aminopeptidase
VP2320	NO	PII uridylyl-transferase
VP2321	NO	hypothetical protein
VP2322	NO	tRNA pseudouridine synthase C
VP2323	NO	hypothetical protein
VP2326	NO	acetyltransferase-like protein
VP2327	NO	hypothetical protein
VP2328	NO	hypothetical protein
VP2331	NO	50S ribosomal protein L31
VP2332	NO	hypothetical protein
VP2333	NO	prolyl-tRNA synthetase
VP2335	NO	hypothetical protein
VP2336	NO	hypothetical protein
VP2338	NO	chitinase
VP2339	NO	hypothetical protein
VP2340	NO	ATP-dependent DNA helicase RecQ
VP2343	NO	hypothetical protein
VP2344	NO	hypothetical protein
VP2345	NO	thiamin biosynthesis lipoprotein ApbE
VP2346	NO	Na(+)-translocating NADH-quinone reductase subunit F
VP2347	NO	Na(+)-translocating NADH-quinone reductase subunit E
VP2348	NO	Na(+)-translocating NADH-quinone reductase subunit D
VP2350	NO	Na(+)-translocating NADH-quinone reductase subunit B
VP2351	NO	Na(+)-translocating NADH-quinone reductase subunit A
VP2352	NO	cell division protein BolA
VP2353	NO	methyltransferase

VP2354	NO	lipoprotein
VP2355	NO	peptidyl-prolyl cis-trans isomerase A
VP2356	NO	AmpG protein
VP2357	NO	transcriptional activator ChrR
VP2358	NO	RNA polymerase sigma factor
VP2365	NO	class V aminotransferase
VP2367	NO	hypothetical protein
VP2368	NO	HesA/MoeB/ThiF family protein
VP2369	NO	murein transglycosylase A
VP2370	NO	hypothetical protein
VP2371	NO	N-acetylglutamate synthase
VP2372	NO	hypothetical protein
VP2373	NO	exodeoxyribonuclease V
VP2375	NO	exonuclease V subunit gamma
VP2377	NO	hypothetical protein
VP2379	NO	DNA damage-inducible gene in SOS regulon, dependent on cyclic AMP and H-NS
VP2380	NO	2,3,4,5-tetrahydropyridine-2,6-carboxylate N-succinyltransferase
VP2381	NO	glycerophosphodiester phosphodiesterase
VP2386	NO	glycerol kinase
VP2387	NO	DeoR family transcriptional regulator
VP2388	NO	glycerol-3-phosphate dehydrogenase
VP2392	NO	hypothetical protein
VP2404	NO	cryptic beta-D-galactosidase subunit beta
VP2406	NO	LysR family transcriptional regulator
VP2407	NO	chromate resistance protein-like protein
VP2431	NO	phosphoserine phosphatase
VP2432	NO	smp protein
VP2433	NO	purine nucleoside phosphorylase
VP2436	NO	deoxyribose-phosphate aldolase
VP2440	NO	peptide chain release factor 3
VP2442	NO	DNA polymerase III subunit psi
VP2448	NO	protease
VP2451	NO	lipoprotein NlpI
VP2458	NO	hypothetical protein
VP2465	NO	RNA-binding protein
VP2468	NO	D-alanyl-D-alanine carboxypeptidase/endopeptidase
VP2470	NO	tyrosyl-tRNA synthetase
VP2471	NO	hypothetical protein
VP2474	NO	iron-sulfur cluster insertion protein ErpA
VP2475	NO	glutamate-1-semialdehyde aminotransferase
VP2476	NO	permease
VP2477	NO	16S rRNA m2G 1207 methyltransferase
VP2478	NO	sensory box sensor histidine kinase/response regulator
VP2479	NO	peptide ABC transporter periplasmic peptide-binding protein
VP2480	NO	peptide ABC transporter permease
VP2482	NO	peptide ABC transporter ATP-binding protein
VP2483	NO	peptide ABC transporter ATP-binding protein
VP2485	NO	N-acetylglucosamine kinase
VP2486	NO	beta-N-hexosaminidase
VP2487	NO	N,N'-diacetylchitobiose phosphorylase
VP2489	NO	iron(III) ABC transporter ATP-binding protein
VP2490	NO	iron(III) ABC transporter permease

VP2491	NO	iron(III) ABC transporter periplasmic iron-compound-binding protein
VP2492	NO	ammonium transporter
VP2493	NO	nitrogen regulatory protein P-II
VP2494	NO	hypothetical protein
VP2500	NO	DnaK suppressor protein
VP2501	NO	hypothetical protein
VP2502	NO	glutamyl-Q tRNA(Asp) synthetase
VP2505	NO	2-amino-4-hydroxy-6- hydroxymethyldihydropteridine pyrophosphokinase
VP2506	NO	3-methyl-2-oxobutanoate hydroxymethyltransferase
VP2507	NO	pantoate--beta-alanine ligase
VP2510	NO	permease
VP2514	NO	carbonic anhydrase
VP2515	NO	hypoxanthine-guanine phosphoribosyltransferase
VP2516	NO	OpaR protein
VP2517	NO	dihydrolipoamide dehydrogenase
VP2519	NO	pyruvate dehydrogenase subunit E1
VP2521	NO	N-acetyl-anhydromuranmyl-L-alanine amidase
VP2525	NO	type IV pilin biogenesis protein PilC
VP2526	NO	type IV prepilin-like proteins leader peptide processing enzyme
VP2527	NO	dephospho-CoA kinase
VP2528	NO	hypothetical protein
VP2530	NO	50S ribosomal protein L19
VP2531	NO	tRNA (guanine-N(1)-)-methyltransferase
VP2532	NO	16S rRNA-processing protein RimM
VP2533	NO	30S ribosomal protein S16
VP2534	NO	signal recognition particle protein
VP2535	NO	hypothetical protein
VP2536	NO	hemolysin
VP2537	NO	S-ribosylhomocysteinase
VP2538	NO	hypothetical protein
VP2539	NO	glutamate--cysteine ligase
VP2540	NO	insulinase family protease
VP2542	NO	quinone oxidoreductase
VP2545	NO	oxaloacetate decarboxylase subunit gamma
VP2546	NO	carbon storage regulator
VP2547	NO	aspartate kinase
VP2548	NO	alanyl-tRNA synthetase
VP2549	NO	recombination regulator RecX
VP2551	NO	CinA-like protein
VP2555	NO	protein-L-isoaspartate O-methyltransferase
VP2556	NO	stationary phase survival protein SurE
VP2557	NO	tRNA pseudouridine synthase D
VP2558	NO	2-C-methyl-D-erythritol 2,4-cyclodiphosphate synthase
VP2559	NO	2-C-methyl-D-erythritol 4-phosphate cytidylyltransferase
VP2560	NO	cell division protein FtsB
VP2562	NO	CTP synthetase
VP2566	NO	23S rRNA 5-methyluridine methyltransferase
VP2568	NO	4'-phosphopantetheinyl transferase
VP2569	NO	pyridoxine 5'-phosphate synthase
VP2571	NO	GTP-binding protein Era
VP2573	NO	signal peptidase I
VP2574	NO	GTP-binding protein LepA

VP2575	NO	sigma-E factor regulatory protein RseC
VP2576	NO	periplasmic negative regulator of sigmaE
VP2577	NO	sigma-E factor negative regulatory protein RseA
VP2578	NO	RNA polymerase sigma factor RpoE
VP2579	NO	hypothetical protein
VP2580	NO	L-aspartate oxidase
VP2581	NO	hypothetical protein
VP2583	NO	hypothetical protein
VP2585	NO	hypothetical protein
VP2586	NO	2-octaprenyl-6-methoxyphenol hydroxylase
VP2587	NO	2-octaprenyl-6-methoxyphenyl hydroxylase
VP2588	NO	hypothetical protein
VP2590	NO	hypothetical protein
VP2592	NO	ribose-5-phosphate isomerase A
VP2593	NO	D-3-phosphoglycerate dehydrogenase
VP2594	NO	hypothetical protein
VP2596	NO	LysE/YggA family protein
VP2598	NO	hypothetical protein
VP2599	NO	fructose-bisphosphate aldolase
VP2600	NO	phosphoglycerate kinase
VP2601	NO	erythrose 4-phosphate dehydrogenase
VP2602	NO	enterobactin receptor protein
VP2603	NO	iron-regulated virulence regulatory protein
VP2606	NO	S-adenosylmethionine synthetase
VP2607	NO	cytochrome c oxidase subunit I
VP2608	NO	hypothetical protein
VP2612	NO	hypothetical protein
VP2613	NO	Holliday junction resolvase-like protein
VP2616	NO	FkuA protein
VP2618	NO	FkuB protein
VP2619	NO	hypothetical protein
VP2620	NO	hypothetical protein
VP2621	NO	deoxyribonucleotide triphosphate pyrophosphatase
VP2623	NO	glutaminase
VP2625	NO	tRNA (guanine-N(7)-)-methyltransferase
VP2627	NO	hypothetical protein
VP2628	NO	membrane-bound lytic murein transglycosylase C
VP2631	NO	HD-GYP domain-containing protein
VP2637	NO	PTS system cellobiose-specific transporter subunit IIB
VP2647	NO	hypothetical protein
VP2648	NO	acetyltransferase
VP2654	NO	aspartate carbamoyltransferase
VP2655	NO	aspartate carbamoyltransferase
VP2656	NO	hypothetical protein
VP2659	NO	BolA/YrbA family protein
VP2661	NO	hypothetical protein
VP2671	NO	sigma-54 modulation protein
VP2673	NO	hypothetical protein
VP2676	NO	peptidase PmbA
VP2677	NO	hypothetical protein
VP2679	NO	ribosomal large subunit pseudouridine synthase A
VP2683	NO	hypothetical protein

VP2684	NO	TldD protein
VP2685	NO	carbon-nitrogen hydrolase
VP2702	NO	MSHA biogenesis protein MshN
VP2706	NO	MSHA biogenesis protein MshJ
VP2710	NO	LuxR family transcriptional regulator
VP2711	NO	UTP-glucose-1-phosphate uridylyltransferase
VP2721	NO	sulfite reductase subunit beta
VP2722	NO	sulfite reductase (NADPH) flavoprotein subunit alpha
VP2723	NO	hypothetical protein
VP2724	NO	hypothetical protein
VP2725	NO	phage shock protein G
VP2729	NO	zinc uptake regulation protein
VP2730	NO	hypothetical protein
VP2731	NO	glucose-6-phosphate isomerase
VP2735	NO	replicative DNA helicase
VP2736	NO	hypothetical protein
VP2737	NO	50S ribosomal protein L9
VP2738	NO	30S ribosomal protein S18
VP2739	NO	primosomal replication protein N
VP2740	NO	30S ribosomal protein S6
VP2741	NO	ribulose-phosphate 3-epimerase
VP2744	NO	3-dehydroquinate synthase
VP2745	NO	shikimate kinase I
VP2748	NO	fimbrial assembly protein PilO
VP2750	NO	fimbrial assembly protein PilM
VP2751	NO	penicillin-binding protein 1A
VP2752	NO	DNA-binding transcriptional regulator OxyR
VP2753	NO	peroxiredoxin family protein/glutaredoxin
VP2755	NO	hypothetical protein
VP2756	NO	bifunctional argininosuccinate lyase/N-acetylglutamate synthase
VP2757	NO	argininosuccinate synthase
VP2758	NO	acetylglutamate kinase
VP2760	NO	acetylornithine deacetylase
VP2761	NO	phosphoenolpyruvate carboxylase
VP2762	NO	hypothetical protein
VP2763	NO	5,10-methylenetetrahydrofolate reductase
VP2764	NO	bifunctional aspartate kinase II/homoserine dehydrogenase II
VP2765	NO	cystathionine gamma-synthase
VP2766	NO	transcriptional repressor protein MetJ
VP2767	NO	malate oxidoreductase
VP2768	NO	bacterioferritin
VP2769	NO	bacterioferritin-associated ferredoxin
VP2772	NO	30S ribosomal protein S7
VP2775	NO	oxidation of intracellular sulfur
VP2776	NO	sulfur transfer complex subunit TusD
VP2777	NO	hypothetical protein
VP2778	NO	FKBP-type peptidylprolyl isomerase
VP2779	NO	hypothetical protein
VP2780	NO	hypothetical protein
VP2781	NO	asparaginase
VP2783	NO	FKBP-type peptidylprolyl isomerase
VP2784	NO	hypothetical protein

VP2787	NO	hypothetical protein
VP2788	NO	ABC transporter ATP-binding protein
VP2789	NO	hypothetical protein
VP2790	NO	hydrolase
VP2791	NO	hypothetical protein
VP2792	NO	phosphoribulokinase
VP2793	NO	cAMP-regulatory protein
VP2794	NO	hypothetical protein
VP2795	NO	succinylglutamic semialdehyde dehydrogenase
VP2796	NO	arginine/ornithine succinyltransferase
VP2797	NO	bifunctional N-succinyldiaminopimelate-aminotransferase/acetylornithine transaminase protein
VP2798	NO	para-aminobenzoate synthase component II
VP2804	NO	tryptophanyl-tRNA synthetase
VP2805	NO	phosphoglycolate phosphatase
VP2806	NO	23S rRNA (guanosine-2'-O-)-methyltransferase
VP2807	NO	ribonuclease R
VP2808	NO	transcriptional repressor NsrR
VP2809	NO	nitric oxide dioxygenase
VP2811	NO	sodium-type polar flagellar protein MotX
VP2812	NO	adenylosuccinate synthetase
VP2813	NO	hypothetical protein
VP2814	NO	HflC protein
VP2815	NO	HflK protein
VP2816	NO	GTPase HflX
VP2817	NO	RNA-binding protein Hfq
VP2818	NO	tRNA delta(2)-isopentenylpyrophosphate transferase
VP2820	NO	N-acetylmuramoyl-L-alanine amidase
VP2822	NO	(Fe-S)-binding protein
VP2823	NO	oligoribonuclease
VP2824	NO	ribosome-associated GTPase
VP2825	NO	phosphatidylserine decarboxylase
VP2826	NO	transporter
VP2827	NO	methyl-accepting chemotaxis protein
VP2828	NO	hypothetical protein
VP2829	NO	phosphoglyceromutase
VP2830	NO	hypothetical protein
VP2831	NO	preprotein translocase subunit SecB
VP2832	NO	NAD(P)H-dependent glycerol-3-phosphate dehydrogenase
VP2833	NO	serine acetyltransferase
VP2834	NO	NlpD-like protein
VP2835	NO	hypothetical protein
VP2836	NO	TetR family transcriptional regulator
VP2837	NO	hypothetical protein
VP2838	NO	lysyl-tRNA synthetase
VP2839	NO	hypothetical protein
VP2840	NO	fumarate reductase flavoprotein subunit
VP2841	NO	fumarate reductase iron-sulfur subunit
VP2842	NO	fumarate reductase subunit C
VP2843	NO	fumarate reductase subunit D
VP2845	NO	elongation factor P
VP2846	NO	hypothetical protein
VP2849	NO	hypothetical protein

VP2850	NO	hypothetical protein
VP2852	NO	co-chaperonin GroES
VP2853	NO	hypothetical protein
VP2855	NO	6-phosphofructokinase
VP2856	NO	ferrous iron efflux protein F
VP2858	NO	transcriptional regulator CpxR
VP2859	NO	two-component sensor protein
VP2861	NO	rRNA methylase
VP2862	NO	FxsA protein
VP2863	NO	aspartate ammonia-lyase
VP2865	NO	thiol:disulfide interchange protein
VP2866	NO	LuxR family transcriptional regulator
VP2867	NO	potassium/proton antiporter
VP2868	NO	hypothetical protein
VP2870	NO	hypothetical protein
VP2872	NO	hypothetical protein
VP2873	NO	fumarate hydratase
VP2875	NO	3-phenylpropionic acid transporter
VP2876	NO	hypothetical protein
VP2877	NO	DNA polymerase III subunit epsilon
VP2881	NO	acetyl-CoA carboxylase biotin carboxylase subunit
VP2883	NO	ribosomal protein L11 methyltransferase
VP2884	NO	NifR3/Smm1 family protein
VP2885	NO	DNA-binding protein Fis
VP2893	NO	DNA-binding transcriptional activator CadC
VP2895	NO	hypothetical protein
VP2896	NO	bifunctional phosphoribosylaminoimidazolecarboxamide formyltransferase/IMP cyclohydrolase
VP2898	NO	phosphoribosylamine--glycine ligase
VP2913	NO	hypothetical protein
VP2916	NO	uroporphyrinogen decarboxylase
VP2920	NO	anti-RNA polymerase sigma 70 factor
VP2925	NO	50S ribosomal protein L1
VP2926	NO	50S ribosomal protein L11
VP2928	NO	preprotein translocase subunit SecE
VP2929	NO	hypothetical protein
VP2936	NO	glutamate racemase
VP2941	NO	DNA-binding transcriptional repressor FabR
VP2942	NO	soluble pyridine nucleotide transhydrogenase
VP2949	NO	chorismate-pyruvate lyase
VP2950	NO	flagellar basal body protein FliL
VP2951	NO	GlpG protein
VP2954	NO	cell division protein FtsX
VP2957	NO	hypothetical protein
VP2958	NO	hypothetical protein
VP2960	NO	hypothetical protein
VP2961	NO	hypothetical protein
VP2964	NO	hypothetical protein
VP2965	NO	hypothetical protein
VP2970	NO	glyceraldehyde-3-phosphate dehydrogenase
VP2971	NO	ArsR family transcriptional regulator
VP2976	NO	hypothetical protein
VP2978	NO	hypothetical protein

VP2983	NO	diaminopimelate epimerase
VP2985	NO	lipoprotein L
VP2986	NO	frataxin-like protein
VP2989	NO	uroporphyrinogen-III synthase
VP2991	NO	HemY protein
VP2999	NO	hypothetical protein
VP3001	NO	thioredoxin
VP3004	NO	hypothetical protein
VP3014	NO	signal peptide protein
VP3015	NO	hypothetical protein
VP3017	NO	transmembrane protein
VP3020	NO	LysR family transcriptional regulator
VP3031	NO	carbonic anhydrase
VP3032	NO	hypothetical protein
VP3033	NO	shikimate 5-dehydrogenase
VP3034	NO	coproporphyrinogen III oxidase
VP3035	NO	Sua5/YciO/YrdC family protein
VP3038	NO	DNA topoisomerase I-like protein
VP3039	NO	hypothetical protein
VP3040	NO	Smf protein
VP3042	NO	peptide deformylase
VP3043	NO	methionyl-tRNA formyltransferase
VP3044	NO	Sun protein
VP3046	NO	potassium uptake protein TrkH
VP3048	NO	hemolysin III
VP3049	NO	SpoOM-like protein
VP3050	NO	hypothetical protein
VP3052	NO	FixG-like protein
VP3054	NO	thiol:disulfide interchange protein
VP3056	NO	periplasmic protein
VP3059	NO	acetolactate synthase 2 regulatory subunit
VP3062	NO	threonine dehydratase
VP3063	NO	DNA-binding transcriptional regulator
VP3068	NO	ATP synthase F0F1 subunit epsilon
VP3069	NO	ATP synthase F0F1 subunit beta
VP3073	NO	ATP synthase F0F1 subunit B
VP3074	NO	ATP synthase F0F1 subunit C
VP3075	NO	ATP synthase F0F1 subunit A
VP3076	NO	F0F1 ATP synthase subunit I
VP3077	NO	ParB family protein
VP3078	NO	ParA family protein
VP3079	NO	16S rRNA methyltransferase GidB
VP3080	NO	tRNA uridine 5-carboxymethylaminomethyl modification enzyme GidA

Table S7 Publicly available *V. mimicus* genomes used in this study

Strain	Size (Mb)	Location(Country)	Isolation_Source
ATCC 33654	4.4387		/
SCCF01	4.48402		/
SX-4	4.27361	China	diarrheal patient
VM603	4.35071		/
VM573	4.36655		/
MB-451	4.31345	Bangladesh: Matlab	diarrheal patient
VM223	4.34797	Brazil: Sao Paulo	/
FDAARGOS_113	4.43209	USA: TN	Human
532-80	4.4325	USA	/
CAIM 602	4.32029		/
CAIM 1882	3.97976		water from a shrimp washing container
CAIM 1883	3.98557		water from a shrimp washing container

Table S8 Antibiotic resistance genes identified from the sequenced strains

Species	Strain Name	Aminoglycoside	Beta-lactam	Fluoroquinolone	Streptogramin	Phenicol	Sulphonamide	Tetracycline	Trimethoprim
V. mimicus	VM14	*aph(3'')-Ib/aph(6)-Id*	-	*qnrVC4*	-	*floR*	*sul2*	*tet(59)*	*dfrA6*
V. mimicus	VM34	*aph(3'')-Ib/aph(6)-Id*	-	*qnrVC4*	-	*floR*	*sul2*	*tet(59)*	*dfrA6*
V. mimicus	VM61	*aph(3'')-Ib/aph(6)-Id*	-	*qnrVC4*	-	*floR*	*sul2*	*tet(59)*	*dfrA6*
V. parahaemolyticus	YK38	-	*blaCARB-47*	-	-	-	-	-	-
V. parahaemolyticus	YK39	-	*blaCARB-47*	-	-	-	-	-	-
V. parahaemolyticus	YK40	-	*blaCARB-47*	-	-	-	-	-	-
V. parahaemolyticus	YK13	-	*blaCARB-48*	-	-	-	-	-	-
V. parahaemolyticus	YK17	-	*blaCARB-47*						
V. parahaemolyticus	YK41	-	*blaCARB-47*	-	-	-	-	-	-
V. mimicus	VM20	*aph(3'')-Ib/aph(6)-Id*	-	*qnrVC4*	-	*floR*	*sul2*	*tet(59)*	*dfrA6*
V. parahaemolyticus	YK45	-	*blaCARB-47*	-	-	-	-	-	-
V. parahaemolyticus	YK51	-	*blaCARB-47*	-	-	-	-	-	-
V. parahaemolyticus	YK49	-	*blaCARB-47*	-	-	-	-	-	-
V. parahaemolyticus	YK32	-	*blaCARB-47*	-	-	-	-	-	*dfrA31*
V. parahaemolyticus	YK33	-	*blaCARB-47*	-	-	-	-	-	-
V. parahaemolyticus	YK34	-	*blaCARB-47*	*qnrC*	-	-	-	-	-
V. scophthalmi	YK47	-	*blaOXA-12*	-	*mph(A)*	*floR*	-	-	-
V. mimicus	VM27	*aph(3'')-Ib/aph(6)-Id*	-	*qnrVC4*	-	*floR*	*sul2*	*tet(59)*	*dfrA6*
V. mimicus	VM37	*aph(3'')-Ib/aph(6)-Id*	-	*qnrVC4*	-	*floR*	*sul2*	*tet(59)*	*dfrA6*
V. mimicus	VM41	*aph(3'')-Ib/aph(6)-Id*	-	*qnrVC4*	-	*floR*	*sul2*	*tet(59)*	*dfrA6*
V. parahaemolyticus	PJ43	-	*blaCARB-47*						
V. parahaemolyticus	PJ42	-	*blaCARB-47*	-	-	-	-	-	-
V. parahaemolyticus	PJ44	-	*blaCARB-47*	-	-	-	-	-	-

V. parahaemolyticus	PJ37	-	*blaCARB-47*	-	-	-	-	-	-
V. parahaemolyticus	PJ18	-	*blaCARB-47*	-	-	-	-	-	-
V. parahaemolyticus	PJ35	-	*blaCARB-47*	-	-	-	-	-	-
V. parahaemolyticus	SH50	-	*blaCARB-47*	-	-	-	-	-	-

Reference

1. Yun, Y. et al. Phylogenetic Analysis of Severe Fever with Thrombocytopenia Syndrome Virus in South Korea and Migratory Bird Routes Between China, South Korea, and Japan. Am. J. Trop. Med. Hyg. **93**, 468 (2015).
2. Nair, G.B. et al. Global dissemination of Vibrio parahaemolyticus serotype O3:K6 and its serovariants. Clin. Microbial. Rev. **20**, 39-48. (2007).
3. Velazquez-Roman, J., León-Sicairos N, de Jesus Hernández-Díaz, L., Canizalez-Roman, A. Pandemic *Vibrio parahaemolyticus* O3:K6 on the American continent. Front. Cell. Infect. Microbial. **3**, 110. (2013).
4. Martinez-Urtaza, et al. Genomic Variation and Evolution of *Vibrio parahaemolyticus* ST36 over the Course of a Transcontinental Epidemic Expansion. Mbio. **8**, pii: e01425-17. (2017).
5. Cowling, D.W., Gardner, I.A., Johnson, W.O. Comparison of methods for estimation of individual level prevalence based on pooled samples. *Prev. Vet. Med.* **39**, 211-25 (1999).
